# *Regulator of Awn Elongation 3*, an E3 ubiquitin ligase, is responsible for loss of awns during African rice domestication

**DOI:** 10.1073/pnas.2207105120

**Published:** 2023-01-17

**Authors:** Kanako Bessho-Uehara, Kengo Masuda, Diane R. Wang, Rosalyn B. Angeles-Shim, Keisuke Obara, Keisuke Nagai, Riri Murase, Shin-ichiro Aoki, Tomoyuki Furuta, Kotaro Miura, Jianzhong Wu, Yoshiyuki Yamagata, Hideshi Yasui, Michael B. Kantar, Atsushi Yoshimura, Takumi Kamura, Susan R. McCouch, Motoyuki Ashikari

**Affiliations:** ^a^Bioscience and Biotechnology Center, Nagoya University, Nagoya, Aichi 464-8601, Japan; ^b^Graduate School of Bioagricultural Sciences, Nagoya University, Nagoya, Aichi 464-8601, Japan; ^c^Plant Breeding and Genetics, Cornell University, Ithaca, NY 14853; ^d^Department of Plant and Soil Science, Texas Tech University, Lubbock, TX 79409; ^e^Graduate School of Science, Nagoya University, Nagoya, Aichi 464-8602, Japan; ^f^Faculty of Biotechnology, Fukui Prefectural University, Eiheiji-Town, Fukui 910-1195, Japan; ^g^Institute of Crop Science, National Agriculture and Food Research Organization, Tsukuba, Ibaraki 305-8634, Japan; ^h^Faculty of Agriculture, Kyushu University, Nishiku, Fukuoka 819-0395, Japan; ^i^Department of Tropical Plant and Soil Sciences, University of Hawaiʻi, Mānoa Honolulu, HI 96822

**Keywords:** African rice, awn, convergent evolution, domestication, E3 ubiquitin ligase

## Abstract

Two species of rice have been independently domesticated from different ancestral wild species in Asia and Africa. Comparison of mutations that underlie phenotypic and physiological alterations associated with domestication traits in these species gives insights into the domestication history of rice in both regions. Asian cultivated rice, *Oryza sativa,* and African cultivated rice, *Oryza glaberrima*, have been modified and improved for common traits beneficial for humans, including erect plant architecture, nonshattering seeds, nonpigmented pericarp, and lack of awns. Independent mutations in orthologous genes associated with these traits have been documented in the two cultivated species. Contrary to this prevailing model, selection for awnlessness targeted different genes in *O. sativa* and *O. glaberrima*. We identify *Regulator of Awn Elongation 3* (*RAE3*) a gene that encodes an E3 ubiquitin ligase and is responsible for the awnless phenotype only in *O. glaberrima*. A 48-bp deletion may disrupt the substrate recognition domain in *RAE3* and diminish awn elongation. Sequencing analysis demonstrated low nucleotide diversity in a ~600-kb region around the derived *rae3* allele on chromosome 6 in *O. glaberrima* compared with its wild progenitor. Identification of *RAE3* sheds light on the molecular mechanism underlying awn development and provides an example of how selection on different genes can confer the same domestication phenotype in Asian and African rice.

## Significance

Selection for a common domestication trait targeted different genes in Asian and African rice. We identify an E3 ubiquitin ligase named *Regulator of Awn Elongation 3* (*RAE3*) that causes awnlessness in African rice and demonstrate its genetic relationship with other genes. Loss of function of *RAE3* leads to awnlessness even when other awn genes (*RAE1* and *RAE2*) are functional; that is, *RAE3* is a key gene for awn elongation in rice. Diversity analysis shows that while the dysfunctional *rae3* allele is fixed across cultivated African rice, it is not found in wild African rice or in Asian rice. The discovery of *RAE3* simultaneously deepens our understanding of awn developmental pathways and lends insight into the complex processes underlying crop domestication.

Crops evolved from wild species through the combined effects of human and natural selection over thousands of years (1). Many of the traits that were selected by humans arose from natural mutations in wild progenitor populations that gave rise to morphological and/or physiological characteristics valued by early hunter-gatherers. Similar traits were often selected in different species across different regions of the world because they contributed to increased ease of harvest, increased yield (i.e., size and/or number of edible fruits, grains, stems or roots), improved palatability, and increased ease of storage. Over time, the accumulation of traits preferred by humans led to crop species that were well differentiated from their wild ancestors. This breeding history is referred to as crop domestication. Crop domestication led to larger and more reliable agricultural harvests, which supported human settlements, population growth, and cultural development. Wheat, maize, and rice are the three most important cereal crops worldwide, together providing approximately 42% of the calories consumed by humans (2). Wheat and maize were each domesticated in a single geographical region (the Middle East and Mexico, respectively), while rice was domesticated independently on two continents, Asia and Africa (2–4).

Asian cultivated rice (*Oryza sativa* L.) was domesticated from the Asian wild rice species, *Oryza rufipogon* around 8,000 to 10,000 y ago (5). African cultivated rice (*Oryza glaberrima* Steud.) was thought to have been domesticated from the African wild rice species, *Oryza barthii,* around 3,000 y ago based on archeological evidence (6, 7). However, recent genome-wide analysis suggests that *O. glaberrima* experienced a prolonged contraction of effective population size (N_e_) beginning as early as 15,000 y ago; this was hypothesized to have resulted from a combination of climatic factors (8) and possibly early low-intensity cultivation (9) (*SI Appendix*, Fig. S1*A*). These parallel rice domestication processes led to convergent phenotypes, such as loss of seed shattering (10, 11), erect plant architecture (12), changes in panicle structure (13), altered pericarp color (14), and reduction in awn length (15, 16). Many genes related to Asian rice domestication have been identified, while less is known about African rice with regard to its origin and domestication history. With the recent increase in genomic information available for African rice, specifically, *O. glaberrima* and its ancestor *O. barthii*, researchers have an expanded set of tools to continue to unravel its domestication history (17–20). Several studies have identified common domestication genes with different causal mutations in *O. sativa* and *O. glaberrima*. In *PROG1*, for example, different mutations in the same gene are responsible for conferring erect plant architecture in Asian and African rice (18). Similarly, different mutations in the *Rc1* gene controlling pericarp color (21) and the *OsSh1* and *Sh4* genes regulating seed shattering (22) were selected in the two domesticated species. These studies demonstrate that the two species have undergone convergent evolution for common domestication traits (17, 18). In other studies focusing on African rice, no selective sweeps were detected around known domestication genes from Asian rice (18, 20). Further, Ndjiondjop et al. (23) identified 37 selective sweeps in *O. glaberrima* that contain no genes previously identified as domestication genes in Asian rice. These findings suggest that many genes underlying domestication and diversification in African rice are yet to be identified. Even in cases where the responsible loci or genes are known, the molecular mechanisms underlying the function of those genes and their contribution to *O. glaberrima* domestication have not yet been analyzed.

**Fig. 1.**
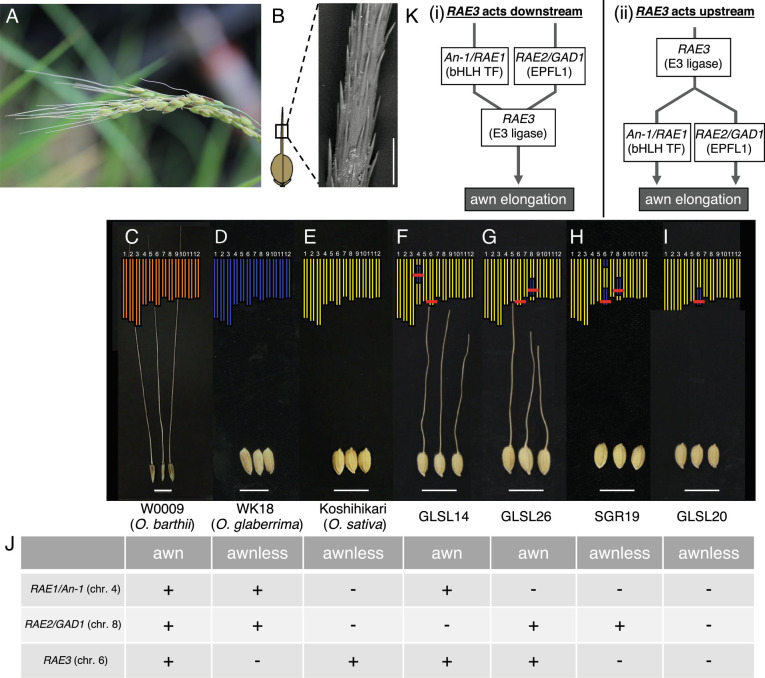
Genetic relationships among *RAE1RAE2*, and *RAE3* in awn elongation. (*A*) Rice panicle with awned seeds. (*B*) The barbed surface of the awn of CSSL, GLSL26. Scale bar represents 200 µm. (*C*–*I*) Phenotypic comparison of awns in cultivated and wild species, and CSSLs carrying functional or dysfunctional *RAE1RAE2,* and *RAE3*. The 12 rectangles above each photo indicate the rice chromosomes and colors correspond to species; orange, *O. barthii*; blue, *O. glaberrima*; yellow, *O. sativa*. Red lines indicate the locus position of *RAE1RAE2,* and *RAE3*. GLSLs are CSSLs carrying a small chromosome segment derived from *O. glaberrima* in *O. sativa* genetic background. SGR19 is backcrossed inbred lines derived from crosses between *O. sativa* and *O. glaberrima*. Detail description is in *Materials and Methods*. Scale bar represents 1 cm. (*J*) Table of awn phenotypes for each line and genotype of *RAE1RAE2,* and *RAE3*. +, functional; −, dysfunctional. (*K*) Graphical representation of genetic relationships among *RAE* genes. bHLH, basic helix–loop–helix; TF, transcription factor; EPFL, epidermal patterning factor like protein; E3 ligase, E3 ubiquitin ligase.

The awn is a needle-like organ that forms at the tip of the seed husk in many grasses (Fig. 1*A*) and is often covered with fine barbs (Fig. 1*B*) (24, 25). The awn assists in seed dispersal through attachment to hairy mammals and protects seeds from feeding damage caused by birds and other wild herbivores (24). In some African rice landraces, awns are maintained because they discourage bird predation, and in areas where birds are particularly problematic, people may rely on awns to ensure a harvest. However, awns are considered a nuisance under agricultural conditions where they hinder agricultural processes such as sowing, harvesting and storage. Therefore, the awnless phenotype has been selected in most areas during the domestication of rice in both Asia and Africa.

To date, several genes regulating awn development in rice have been identified, including *An-1*, also annotated as *RAE1*, which encodes a bHLH transcription factor (15, 26); *RAE2*, also annotated as *GAD1*, which encodes a secreted signal peptide EPFL (16, 27); *LABA1*, which encodes a cytokinin activating enzyme (25) for regulating awn surface structure; and *OsETT2* and *DL*, which are both transcription factors (28). Among these genes, loss of function of *An-1/RAE1* and *RAE2/GAD1* are mainly responsible for the loss (or shortening) of awn during Asian rice domestication according to quantitative trait locus (QTL) and chromosome segment substitution line (CSSL) analyses (29, 30). However, each of these studies focused exclusively on Asian rice, and the role of these genes in awn loss in African rice has not been examined.

African cultivated rice (*O. glaberrima*) usually lacks awns, whereas its ancestral wild species (*O. barthii*) possesses long awns (Fig. 1 *C* and *D*). Despite the fact that most *O. glaberrima* does not form awns, it retains functional forms of *An-1/RAE1* and *RAE2/GAD1* based on our previous analyses (16, 26). This finding suggests that the loss of awns in *O. glaberrima* is due to mutation in gene(s) other than *An-1/RAE1* and *RAE2/GAD1*. Our previous study (26) showed that a single locus on the long arm of chromosome 6 in *O. glaberrima* is responsible for the loss of awns in African rice. This locus was designated as *Regulator of Awn Elongation 3* (*RAE3*) (*SI Appendix*, Fig. S1*B*). No previous QTL or gene mapping study had identified a locus associated with awn length in this region of chromosome 6 (31–33). This is likely due to the fact that the genetic background of the materials used in previous QTL or CSSL studies were *O. sativa*, where a functional *RAE3* gene masks its contribution to the awn phenotype.

In this study, we identified *RAE3* as a gene responsible for awnlessness in African rice and revealed that *RAE3* encodes a protein with a RING-H2 domain. Proteins with RING-H2 domains have been reported to function as E3 ubiquitin ligases in animals, plants, and eukaryotic microorganisms (34–36). E3 ubiquitin ligase, in combination with E1 and E2 ubiquitin-conjugating enzymes, regulates the ubiquitination of substrates. Ubiquitination leads to several functional outcomes in a reversible modification manner. First, it is a marker of proteasome-dependent degradation and is involved in protein quality control, protein reuse, and signal transduction. Specifically, in plant hormone signaling pathways, ubiquitination contributes to the degradation of signal repressors such as Aux/indoleacetic acid (IAA) (37) and DELLA (38) via the 26S-proteasome. Secondly, in a proteasome independent manner, ubiquitination is involved in protein cell localization. Polyubiquitination of the BRI1 receptor, which normally localizes to the plasma membrane, changes its localization to an endosomal compartment (39). Lastly, ubiquitination remodels the surface of substrate proteins thereby potentially affecting protein stability and interactions with other proteins (40).

Here, a 48-bp deletion in the C-terminal region of *RAE3* was detected in *O. glaberrima* (hereafter referred to as the *Ograe3* allele), whereas this deletion was not found in *O. barthii* or in *O. sativa*. This deletion resulted in a frameshift that is predicted to modify the protein structure of RAE3 and affect its capacity to bind with its substrates. Comparison of *RAE3* sequences in diversity panels of African rice revealed that the loss of function conferred by *Ograe3* was selected during the domestication of African rice. Our results suggest that independent gene selection led to convergent evolution of awnlessness during Asian and African rice domestication.

## Results

### RAE3 May Function Cooperatively with RAE1 or RAE2 in Rice Awn Elongation.

Long awns are observed in *O. barthii* accessions that contain functional alleles at *RAE1* and *RAE2*, while an awnless phenotype is observed in *O. glaberrima* accessions carrying the same functional *RAE1* and *RAE2* alleles (hereafter referred to as *OgRAE1* and *OgRAE2*) (16, 26) (Fig. 1 *C*, *D*, and *J*). We hypothesize that a third gene, *RAE3,* that impacts the function of *RAE1* and *RAE2* was selected during African rice domestication (*SI Appendix*, Fig. S1*B*). To identify *RAE3,* the gene responsible for the awnless phenotype in African rice, we compared awn phenotypes and genotypes of wild, cultivated, and several CSSLs designated GLSL (41). GLSLs carried small segments of *O. glaberrima* chromosomes in an *O. sativa* (cv. Koshihikari) genetic background. *O. sativa* carried dysfunctional *rae1* and *rae2* alleles (hereafter referred to as *Osrae1* and *Osrae2*), along with a functional *RAE3* allele (*OsRAE3*) and presented an awnless phenotype (Fig. 1 *E* and *J*). Two GLSL lines, GLSL14 and GLSL26, presented awned phenotypes (Fig. 1 *F*, *G*, and *J*). GLSL14 carried the functional *OgRAE1* (chromosome 4), and GLSL26 carried the functional *OgRAE2* (chromosome 8). Since the genetic background of GLSL is *O. sativa*, both GLSL lines carried functional *OsRAE3* alleles on chromosome 6. These observations confirmed that *OgRAE1* and *OgRAE2* are both functional alleles and that they may individually coordinate with *OsRAE3* to elongate the awns. SGR19, which carries *OgRAE2* and *Ograe3* regions from *O. glaberrima* in *O. sativa* cv. Taichung65 (T65) genetic background had no awns (Fig. 1 *H* and *J*). This demonstrates that a dysfunctional *Ograe3* allele masks the function of *OgRAE2*. Considering the genotype of 3 *RAE* genes and awn phenotype of *O. glaberrima* and selected CSSLs, a dysfunctional *rae3* allele from *O. glaberrima* is sufficient to mask the function of *OgRAE1* and *OgRAE2*. In addition, GLSL20, which carries *Ograe3* on chromosome 6 in the Koshihikari (*O. sativa*) genetic background, has 3 loss of functional alleles of *RAE* genes (*rae1*, *rae2* and *rae3*) and show awnless phenotype (Fig. 1 *I* and *J*). Based on these observations, we propose that a pair of functional genes, either *RAE1* + *RAE3* or *RAE2* + *RAE3,* is necessary to promote awn elongation, whereas a single functional allele at *RAE1*, *RAE2,* or *RAE3* is unable to do so. Together, these results suggest that *RAE3* functions as a hub gene, meaning that it connects two genes from independent pathways, to promote awn elongation in rice, although it remains unknown whether *RAE3* is located downstream or upstream of *RAE1* and *RAE2* (Fig. 1*K*).

### Mapping of RAE3 as the Gene Responsible for Awn Elongation in African Rice.

**Fig. 2.**
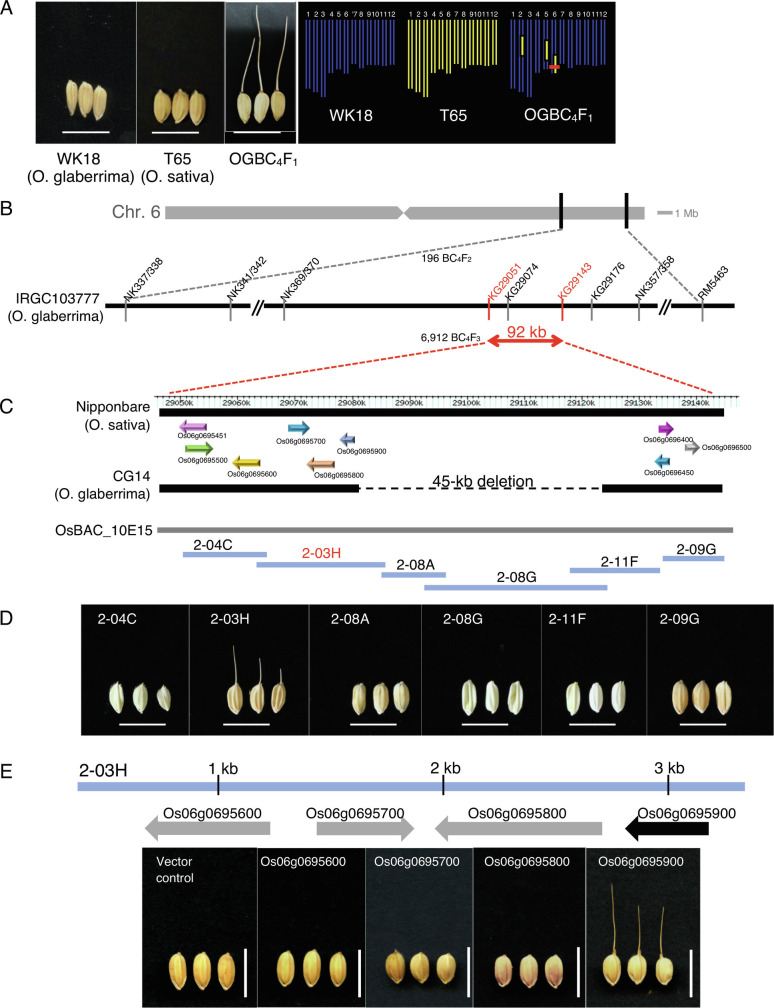
Map-based cloning and identification of *RAE3*. (*A*) Comparison of awn phenotypes among WK18 (*O. glaberrima*), T65 (*O. sativa*), and OGBC_4_F_1_ (backcrossed lines of T65 and *O. glaberrima*). The 12 rectangles in right panes indicate the rice chromosomes and colors correspond to species; blue, *O. glaberrima*; yellow, *O. sativa*. Bar = 1 cm. Red bar represents *RAE3* location. (*B*) *RAE3* was further delimited to a 92-kb genomic region between markers KG29051 and KG29143, shown in red. (*C*) Comparison of genome sequences and annotation of Nipponbare (*O. sativa*) and CG14 (*O. glaberrima*). Black bars represent genomic sequences of Nipponbare and CG14, respectively. Arrows represent the predicted open reading frames according to RAP-DB. The gray bar represents the sequence range of *O. sativa* BAC, OsBAC_10E15 and blue bars indicate sub-clones derived from OsBAC_10E15. Highlighting of 2-03H in red indicates that transformants carrying this subclone complemented the awn phenotype. (*D*) Seed phenotypes of transgenic lines carrying subclones from OsBAC_10E15. (*E*) Awn phenotypes of transgenic plants with each candidate gene showing as gray and black arrows on the 2-03H subclone plasmid. Scale bars represent 1 cm.

To map *RAE3* on chromosome 6, we first produced 100 OGBC_4_F_1_ lines carrying chromosome segments of *O. sativa* cv. T65 in the *O. glaberrima* genetic background. Among these 100 lines of OGBC_4_F_1_, only one OGBC_4_F_1_ line that was heterozygous for regions on chromosomes 2, 5, and 6 showed an awned phenotype (Fig. 2*A*). Its progeny, designated OGBC_4_F_2_, segregated into awned and awnless phenotypes with a 3:1 ratio (26). From this, we concluded that a recessive allele of *rae3* in *O. glaberrima* caused the awnless phenotype. To identify the functional *RAE3* allele in *O. sativa* that induces awn elongation, we undertook positional cloning with approximately 7,000 lines of OGBC_4_F_3_. Genetic linkage analysis delimited the candidate region to within about 92 kb between markers KG29051 and KG29143 on chromosome 6 (Fig. 2*B*). Comparison of the genomic structure of this region between *O. sativa* cv. Nipponbare and *O. glaberrima* cv. CG14 revealed that *O. glaberrima* has many mutations and a 45-kb deletion in the candidate region (Fig. 2*C*). *O. barthii,* which has awns, does not have the 45-kb deletion found in *O. glaberrima* (*SI Appendix*, Fig. S2). According to the Rice Annotation Database (RAP-DB, https://rapdb.dna.affrc.go.jp, searched on February 4, 2021), no predicted genes were present in the region of the *O. sativa* cv. Nipponbare genome corresponding to the 45-kb deletion, while 9 genes were predicted in the 47-kb region flanking the deletion (Fig. 2*C*). We screened an *O. sativa* bacterial artificial chromosome (BAC) library and identified a clone, OsBAC_10E15, which included the entire 92-kb candidate region (Fig. 2*C*). Six sub-clones retrieved from OsBAC_10E15 were introduced into SGR19, a line carrying *O. glaberrima* substitutions on chromosomes 8 and 6, including a functional *OgRAE2* and a dysfunctional *Ograe3* allele (*SI Appendix*, Fig. S3*A*). Comparison of CSSLs (Fig. 1 *F*–*I*) indicated that *RAE1* and *RAE2* function independently, while *RAE3* appears to act in combination with *RAE1* and/or *RAE2*; thus, a functional variant of either *RAE1* or *RAE2* is needed for the *RAE3* complementation test. Introduction of subclone 2-03H into SGR19 recovered the awned phenotype in transgenic lines (Fig. 2*D* and *SI Appendix*, Table S1). Four genes, *Os06g0695600*, *Os06g0695700*, *Os06g0695800*, and *Os06g0695900,* were included in the 2-03H clone. To analyze these genes, overexpression constructs of each gene were transformed into SGR19. Among these, only the overexpression line *Os06g0695900* complemented the awned phenotype (Fig. 2*E* and *SI Appendix*, Fig. S3 *B*–*D* and Table S1). Together, these results indicated that *Os06g0695900* is *OsRAE3* and regulates awn elongation.

### RAE3 Expression Pattern in *O. sativa*.

To elucidate the function of *OsRAE3*, its expression pattern was examined in several organs of *O. sativa* cv. Nipponbare using the Rice Expression Database [RED (42); http://expression.ic4r.org, searched on January 4, 2020] based on RNA-seq data. We retrieved the expression values and calculated the mean value for each tissue; anther, callus, leaf, panicle, root, seed, and shoot. *OsRAE3* (*Os06g0695900*) expression was highest in panicles and anthers and lowest in vegetative organs such as the leaf, root, and shoot (*SI Appendix*, Fig. S4*A*). There is a possibility that the expression level of *RAE3* may be altered due to the existence of functional *RAE1* and *RAE2,* as indicated in Fig. 1 *K*, *i*. If *RAE1* and *RAE2* contributed to *RAE3* expression as indicating in Fig. 1 *K*, *i*, the expression level of *RAE3* should be up-regulated in GLSL14 or GLSL26 compared with GLSL20 or Koshihikari. We performed quantitative expression analysis of *RAE3* using GLSL14 (Fig. 1*F*) and GLSL26 (Fig. 1*G*), which carry functional *OgRAE1* and *OgRAE2* with functional *OsRAE3,* and GLSL20 (Fig. 1*I*), which carries a dysfunctional *Ograe3* in the *O. sativa* genetic background. As first, we observed that expression of *OgRAE1* and *OgRAE2* in GLSL14 and GLSL26. GLSL14 and GLSL26 showed higher expression levels of *OgRAE1* and *OgRAE2* compared with the other lines (*SI Appendix*, Fig. S4 *B*, *i* and *ii*), consistent with previous results (15, 16). In contrast, the expression level of *RAE3* showed no significant differences among the samples carrying different combinations of functional *RAE1/RAE2* and *RAE3* (*SI Appendix*, Fig. S4 *B*, *iii*). This result suggests that *RAE3* may not be transcriptionally regulated by *RAE1* or *RAE2*.

### RAE3 Sequence Comparison between *O. glaberrima* and *O. sativa*.

To identify the mutations responsible for loss of function of *RAE3* in *O. glaberrima*, we compared the sequences of *O. glaberrima* with its ancestral species, *O. barthii*, which is expected to retain a functional *RAE3* allele for awn elongation (*SI Appendix*, Fig. S5*A*). Several single-nucleotide polymorphisms (SNPs) were detected in the promoter and terminator regions of the gene, which exhibited 96% and 97% identity, respectively, between *O. glaberrima* and *O. barthii*. In addition, a 48-bp deletion that included the stop codon of *Ograe3* and resulted in extension of the coding region was detected in *O. glaberrima* (*SI Appendix*, Fig. S5*A*). The 48-bp deletion was not present in *O. sativa* or in *O. barthii,* both of which carry functional *RAE3* alleles and have very similar amino acid sequence (*SI Appendix*, Fig. S5*B*). Some nonsynonymous SNPs were detected in the coding region in *O. glaberrima* compared with *O. sativa*, but as these SNPs were shared with *O. barthii,* they were determined not to be related to the loss of function of RAE3. This suggests that the 48-bp deletion may be responsible for the loss of *RAE3* function in *O. glaberrima*.

### RAE3 Encodes an E3 Ubiquitin Ligase and Localizes to the Cell Membrane.

Based on amino acid sequence analysis, *RAE3* is predicted to encode a protein with a zinc finger RING-H2 domain. The phylogenetic tree of *RAE3* gene homologs shows that they can be classified into two clades associated with monocots and dicots; *RAE3* is classified into the monocot clade (*SI Appendix*, Fig. S6*A*). The hydrophobic region, GLD motif, and RING-H2 domain are conserved among diverse plant species, while other domains are not well conserved (*SI Appendix*, Fig. S6*B*). One of the closest homologs of *RAE3* in *Arabidopsis thaliana* is *At5G05810* (*SI Appendix*, Fig. S6*A*), named *ATL43*, which has been reported to be involved in abscisic acid signal transduction (43). The ATL family consists of 80 genes in *Arabidopsis* and 121 genes in rice (43). ATLs are comprised of three well-conserved regions: the N-terminal hydrophobic region, a GLD motif region, and a RING-H2 domain (*SI Appendix*, Fig. S7*A*). The N-terminal hydrophobic region is a common structure in transmembrane proteins, and all ATL family proteins analyzed to date are localized to the plasma membrane, ER membrane, or thylakoid membrane (44–46). The RING-H2 domain was shown to bind directly to the E2 ubiquitin-conjugating enzyme in *EL5* (47). The GLD motif has been suggested to play a role in regulating the binding of E2 enzymes, but its precise function remains unclear (48). In addition, the C-terminal region, which follows the RING-H2 domain, has relatively high diversity within this gene family (*SI Appendix*, Fig. S6*B*) and is thought to be a substrate recognition domain (43). *Arabidopsis ATL31* was reported to bind to its substrate, 14-3-3 protein, via the C-terminal region (49).

To examine the subcellular localization of *RAE3*, we made a YFP fusion construct and firstly observed its subcellular localization through bombardment in onion epidermal cells. Microscopic observation revealed that OsRAE3 was localized to the plasma membrane and to small particles in the cytosol that colocalized with FM4-64, a membrane marker (*SI Appendix*, Fig. S7*B*). We also observed the subcellular localization of RAE3 in rice protoplasts. Fluorescence from OsRAE3-YFP was observed at the plasma membrane (*SI Appendix*, Fig. S7*C*). Further, the 3D structure predicted by AlphaFold2 (50) indicated that OsRAE3 contains a transmembrane domain near the N-terminal region (*SI Appendix*, Fig. S7*D*). Taken together, these results provide evidence that OsRAE3 localizes at the plasma membrane and is expected to play a role as an E3 ubiquitin ligase. That is, OsRAE3 may regulate awn elongation by attaching ubiquitin to its substrates involved in signal transduction of awn elongation for degrading, change their localization, or remodeling surface feature (*SI Appendix*, Fig. S7*E*).

### Auxin Degron System Indicates that RAE3 Has an E3 Ubiquitin Ligase Function.

The auxin-inducible degron (AID) system was developed as a tool for rapid and inducible protein degradation in nonplant systems (51), such as yeast. TIR1, an E3 ubiquitin ligase that is a key component of auxin signaling, binds to IAA17 in the presence of auxin; IAA17 is ubiquitinated by TIR1 and degraded via the 26S proteasome pathway (51). Using the AID system, the ability of E3 ubiquitin ligase can be evaluated based on the degree of degradation of IAA17.

To examine the ability of OsRAE3 to function as an E3 ubiquitin ligase, we employed the AID system using a chimeric protein fused with OsTIR1 (Os04g0395600). If OsRAE3 has E3 ubiquitin ligase activity, the chimeric protein is expected to interact with the E2 enzyme via the RAE3-derived RING-H2 domain and induce ubiquitination, resulting in the degradation of IAA17 (*SI Appendix*, Fig. S8*A*). First, we constructed a plasmid of *OsRAE3* in which the C-terminal substrate recognition domain was swapped with that of *OsTIR1* (*SI Appendix*, Fig. S8 *B* and *C*). As the hydrophobic domain in the N-terminal region of the ATL protein may be toxic to yeast (52), it was removed from the *OsRAE3* construct (*SI Appendix*, Fig. S8*C*) which is referred to as FLAG-ΔRAE3(WT)-TIR. According to previous research, substitution of one of the predicted Zn binding residues in the RING-H2 domain causes loss of E3 ubiquitin ligase function of the ATL protein (49, 52). We established a mutated version of *OsRAE3* with a serine substitution of the 136th cysteine, designated FLAG-ΔRAE3(C136S)-TIR (*SI Appendix*, Fig. S8*D*). Both constructs, FLAG-ΔRAE3(WT)-TIR and FLAG-ΔRAE3(C136S)-TIR, were expressed in yeast and the expression levels were confirmed through western blotting with anti-Flag antibody (*SI Appendix*, Fig. S8*E*).

**Fig. 3.**
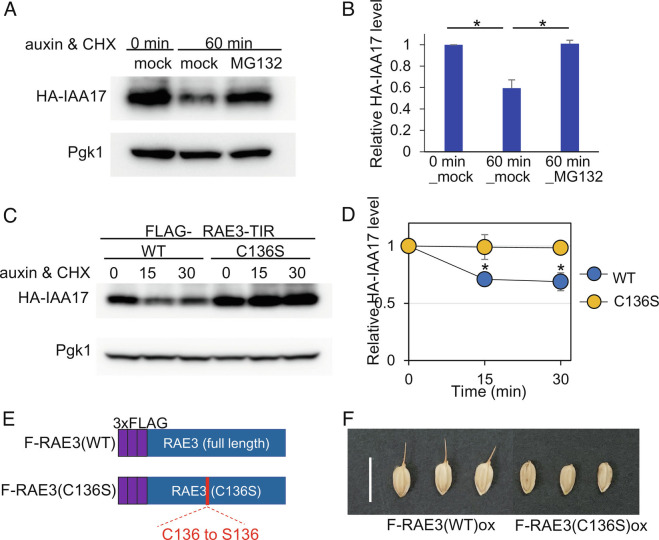
RAE3 functions as a E3 ubiquitin ligase to degrade its substrates. (*A*) *RAE3* activity assay using the AID system. HA-IAA and Pgk1 (loading control) were detected through immunoblotting using antibodies to HA and to Pgk1, respectively. Yeast strains expressing HA-IAA and FLAG-ΔRAE3(WT)-TIR were initially grown in synthetic medium lacking leucine and uracil for 1 h before addition of 300 μM auxin. (*B*) Quantitative results of the RAE3 activity assay shown in *A*. Y-axis indicates relative HA-IAA17 protein accumulation compared with 0-min_mock, which was set to 1. (*C*) HA-IAA17 protein level detected by RAE3 activity assay using the AID system with FLAG-ΔRAE3(WT)-TIR or FLAG-ΔRAE3(C136S)-TIR carrying a point mutation in the RING-H2 domain. The antibodies used are described above for *A*. (*D*) Quantitative results of the RAE3 activity assay shown in *C*. Y-axis indicates HA-IAA17 protein accumulation relative to 0-min_mock, which was set to 1. Blue and orange indicate FLAG-ΔRAE3(WT)-TIR and FLAG-ΔRAE3(C136S)-TIR, respectively. The entire experiment was replicated three times and scale bars represent SD. Significance was tested by one-way analysis of variance (ANOVA) with Tukey’s comparison. *P <0.05. (*E*) Schematic image of F(FLAG)-RAE3(WT) and F-RAE3(C136S) constructs. (*F*) Awn phenotypes of transgenic plants carrying FLAG-RAE3(WT) or FLAG-RAE3(C136S) in the SGR19 background. Scale bar represents 1 cm.

For AID system analysis, the FLAG-ΔRAE3(WT)-TIR construct and HA-IAA17 construct were introduced together into a yeast strain YTK2812, and then incubated with 300 µM auxin and 200 µg/ml cycloheximide (CHX), which is a translation inhibitor (Fig. 3*A*). HA-IAA17 accumulation was observed in the absence of auxin in transgenic yeast with the FLAG-ΔRAE3(WT)-TIR construct (Fig. 3*A*). At 60 min after the addition of auxin and CHX, IAA17 was significantly degraded compared with the absence of auxin condition, whereas its degradation was repressed by the addition of MG132, which is a 26S proteasome inhibitor (Fig. 3 *A* and *B*). According to this result, we concluded that the OsRAE3 protein works as an E3 ubiquitin ligase. FLAG-ΔRAE3(WT)-TIR protein was slightly degraded with CHX treatment and its degradation was repressed by MG132 treatment (*SI Appendix*, Fig. S9 *A* and *B*), suggesting self-ubiquitination of the FLAG-ΔRAE3(WT)-TIR protein and degradation by the 26S proteasome. In the transgenic yeast line with FLAG-ΔRAE3(WT)-TIR, the degradation of the substrate HA-IAA17 depends on the time since the addition of auxin and CHX (Fig. 3 *C* and *D*). In contrast, the transgenic line with FLAG-ΔRAE3(C136S)-TIR exhibited no decrease in HA-IAA17 protein (Fig. 3 *C* and *D*). The amount of FLAG-ΔRAE3(WT)-TIR protein decreased over time with CHX treatment, whereas no decrease in the amount of FLAG-ΔRAE3(C136S)-TIR protein was observed (*SI Appendix*, Fig. S9 *C* and *D*). We also observed that the RAE3 protein without the hydrophobic region fused with GFP (ΔRAE3(WT)-TIR-GFP) was localized at cytoplasm in yeast and the mutated OsRAE3 chimeric protein (ΔRAE3(C136S)-TIR1-GFP) showed the same localization pattern in yeast cells (*SI Appendix*, Fig. S9*E*). The fluorescence intensity was stronger in ΔRAE3(C136S)-TIR1-GFP than in ΔRAE3(WT)-TIR-GFP, corresponding to the results of western blotting. Taken together, these results suggest that the point mutation of the RING-H2 domain impairs the E3 ubiquitin ligase activity of OsRAE3, preventing both substrate ubiquitination and self-ubiquitination of the OsRAE3 protein, and providing evidence of RAE3’s E3 ubiquitin ligase potential in vivo.

### Mutation of the RING-H2 Domain of OsRAE3 Negates Its Awn Elongation Function.

To clarify the function of *RAE3* in awn development *in planta*, we evaluated lines overexpressing two variants in the SGR19 background (*SI Appendix*, Fig. S3*A*). Full-length *OsRAE3* fused with 3×FLAG was designated FLAG-RAE3(WT)ox, and the construct with a point mutation in the RING-H2 domain of full-length *OsRAE3* was called FLAG-RAE3(C136S)ox (Fig. 3*E*). Awn elongation was observed in the transgenic lines of FLAG-RAE3(WT)ox, but not in FLAG-RAE3(C136S)ox lines (Fig. 3*F* and *SI Appendix*, Table S1). According to the fluorescence observation of OsRAE3(WT)-YFP and OsRAE3(C136S)-YFP in rice protoplasts, both are localized at the plasma membrane (*SI Appendix*, Fig. S7*C*). This result indicates that the point mutation in the RING-H2 domain of RAE3 does not affect the subcellular localization of RAE3. In total, the loss of function of an E3 ubiquitin ligase due to mutation of the RING-H2 domain resulted in loss of *RAE3* activity as a positive regulator of awn elongation in rice.

### Loss of Function of RAE3 Was Selected during African Rice Domestication.

**Fig. 4.**
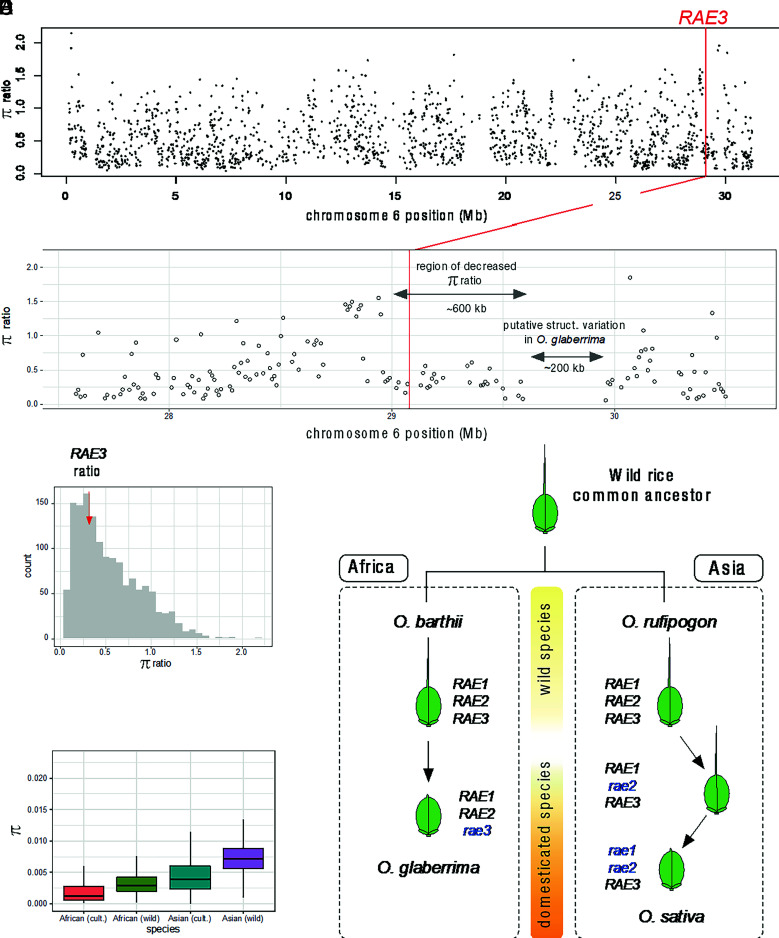
Nucleotide diversity analysis of cultivated and wild African rice. Nucleotide diversity ratios (πglaberrima/ πbarthii) across chromosome 6 (*A*) and across an approximately 3-Mb region around *RAE3* (*B*). Nucleotide diversity was computed in 10-kb bins for all 10-kb windows with a bin-average SNP missing data rate < 20%. Each point in represents the π ratio value for a single 10-kb window. Red vertical line indicates the position of the 48-bp indel of *RAE3* in *A* and *B*. Gray box denotes region of depressed nucleotide diversity ratio values in *B*. (*C*) Distribution of nucleotide diversity (π) ratios across chromosome 6 for the 10-kb windows shown in panel *B*. “count” indicates the number of regions that have a certain nucleotide diversity ratio as indicated within each bin. The average *RAE3* nucleotide diversity ratio (computed using 50-SNP sliding windows with a 2-SNP step size) is marked with a red vertical arrow; this corresponds to the 34th percentile of the distribution. All positions are based on the MSUv7 annotation. (*D*) Distribution of nucleotide diversity across chromosome 6 in wild and cultivated African and Asian rice groups. (*E*) Proposed model of convergent evolution of the awnless phenotype in two rice domestication regions, Asia and Africa.

To test for a selective sweep in the region of *Ograe3*, we further examined resequencing data from diverse accessions of *O. glaberrima* (n = 120) and *O. barthii* (n = 62), originating from several locations, including around the Niger River where African rice domestication occurred (6, 19, 20) (*SI Appendix*, Fig. S10*A* and Table S2). Nucleotide diversity (π) of *O. glaberrima* was estimated relative to that of *O. barthii* in 10-kb window bins across chromosome 6 (Fig. 4*A*). Nucleotide diversity ratios (π ratios; π of *O. glaberrima* to π of *O. barthii*) were mostly below 1.0, as expected in comparisons of a cultivated species with its wild relative. The region surrounding *RAE3* was characterized by a drop in the π ratio, followed by a plateau that extends ~600-kb downstream (Fig. 4*B*). Beyond the plateau, we discovered a ~200-kb region that had systematic missing data in ~46% of *O. glaberrima* accessions; this pattern is consistent with structural variation. Examining nucleotide diversity of *RAE3* itself, we found that π in *O. glaberrima* was about one-third that of *O. barthii* (average, 0.315) (Fig. 4*B*), and that the gene-based π ratio corresponded to the 34th percentile of the π ratio distribution on chromosome 6 (Fig. 4*C*). This was higher than what would be expected after a hard selective sweep; however, other pieces of evidence supported selection at *RAE3*. Haplotype analysis revealed three haplotypes (H1, H2, and H3) at *RAE3*, with 95% of *O. glaberrima* carrying H1, the haplotype that harbored the 48-bp deletion in the *RAE3* coding sequence (CDS) region. In contrast, 91% of *O. barthii* accessions had the H2 haplotype, which did not carry the deletion (*SI Appendix*, Fig. S10*B* and Dataset S1). In addition, all *O. sativa* accessions had H3, which also did not have the 48-bp deletion. Of the five *O. glaberrima* accessions that carried the nondeletion (functional) allele, and one *O. barthii* accession that carried the deletion (dysfunctional) allele, haplotype analysis showed that these are likely due to introgressions between the wild and cultivated gene pools (*SI Appendix*, Fig. S10*C*), indicating that the dysfunctional allele is fixed in *O. glaberrima*. Interestingly, all five *O. glaberrima* accessions with the nondeletion allele originated from the Senegal (n = 3)/Guinea-Bissau (n = 2) region (*SI Appendix*, Fig. S10*A*).

To clarify the relationship between the 48-bp indel in *RAE3* and the awn phenotype, we evaluated lines of *O. barthii* (n = 8) and *O. glaberrima* (n = 8) that differed in awn phenotype but were known to carry functional alleles at the *RAE2* locus (16). First, we designed two PCR primers (KB119 and KB70) for the proximal and distal regions of the 48-bp deletion in *RAE3*, and one primer (KB112) for the interior of the 48-bp deletion (*SI Appendix*, Fig. S11*A*). The band sizes amplified with each primer combination are shown in *SI Appendix*, Fig. S11*B*. Amplification with KB119-KB70 was observed in all samples, and all amplicons in *O. glaberrima* were shorter than in *O. barthii* (*SI Appendix*, Fig. S11*C*). When the samples were amplified with KB119-KB112, no amplification occurred in *O. glaberrima*, whereas amplification was observed in all *O. barthii* samples. In this set, the *O. glaberrima* samples all had the awnless phenotype and the *O. barthii* samples were fully fixed for the awned phenotype (*SI Appendix*, Fig. S11*C*). Given that all 16 lines carried a functional *RAE2* (16), this result supports the conclusion that the 48-bp indel is the causative functional nucleotide polymorphism for the awnless phenotype in *O. glaberrima*.

## Discussion

In this study, we identified *RAE3,* which encodes a RING type E3 ubiquitin ligase, as a gene for awn elongation. The genes encoding E3 ubiquitin ligase are highly diverse, including at least 600 genes in humans (53) and 1,300 genes in plants (54). E3 ubiquitin ligases that function in organogenesis and size regulation have been reported in various plants. For example, *Arabidopsis XBAT32* ubiquitinates the aminocyclopropane-1-carboxylic acid synthases *ACS4* and *ACS7*, which are involved in ethylene biosynthesis, and causes positive regulation of lateral root formation (55). The E3 ubiquitin ligase *GW2* directly interacts with *EXPLA1* (*expansin-like 1*) to ubiquitinate and degrade it, influencing rice grain size (56). Thus, temporal and spatial regulation of substrate levels by E3 ubiquitin ligases are involved in a variety of mechanisms that affect plant morphogenesis and organ size. Based on other ATL family proteins which are homologs of *RAE3*, the substrate recognition site was predicted to be in the C-terminal region (43, 49). Consistent with this expectation, extension of the amino acid sequence in Ograe3 may lead to loss of function via probable effects on substrate binding. We observed that the C-terminal region of *RAE3* is conserved across the monocot subclade in this family as shown in *SI Appendix*, Fig. S6*B*, and thus a change in protein conformation or physical or charge disorder might affect the binding of Ograe3 to its substrates in *O. glaberrima*. Further, the mutated version of *OsRAE3* was not able to complement the awn phenotype in SGR19. That is, the point mutation in RING-H2 domain caused loss of function of *OsRAE3* as an E3 ubiquitin ligase and repressed awn elongation. The mutation in the RING-H2 domain, however, does not affect subcellular localization of RAE3 itself. These results support the hypothesis that the functional form of *RAE3* has the capacity to degrade proteins or, to alter subcellular localization or protein surface of substrates that negatively regulate awn elongation (*SI Appendix*, Fig. S12). Determining the substrates of OsRAE3 that would be the suppressor will deepen our understanding of the comprehensive molecular mechanism underlying awn elongation.

According to the results of genetic analysis, *RAE1* and *RAE2* act in concert with *RAE3*. Expression patterns of these genes overlap strongly in the young panicle and anther (15, 16), and these spatiotemporal expression patterns suggest that *RAE1*, *RAE2,* and *RAE3* function cooperatively. Expression levels of *RAE3* in several CSSLs harboring functional *RAE1* or *RAE2* alleles suggest that *RAE3* may not be transcriptionally regulated by *RAE1* or *RAE2* (*SI Appendix*, Fig. S4*B*) as suggesting in Fig. 1 *K*, *i*. To prove the second possibility indicated in Fig. 1 *K*, *ii*, we need to examine pyramided lines carrying *RAE1-rae3* or *RAE2-rae3* combinations. While it is obvious that *RAE1–RAE2–RAE3* genetically regulate awn elongation, molecular network is still unclear. A recent study in sorghum identified the *SbAWN1* gene encoding a transcription factor with an ALOG domain that is responsible for awn loss during sorghum domestication (57). This transcription factor binds directly to the regulatory regions of the homologs of *DL* (28) and *LKS2* (58), which are genes responsible for awn elongation in rice and barley, respectively, and downregulates the expression of these genes, resulting in an awnless phenotype in sorghum. Revealing the relationship between *RAE3, OsAWN1* and other awn-related genes in rice will enhance our understanding of the signal transduction pathways regulating awn elongation. Similarly, discovery of yet unidentified genes will likely fill in the missing link(s) in the *RAE1–RAE2–RAE3* signaling network.

The 48-bp deletion in the C-terminal region of *RAE3*, which is fixed in *O. glaberrima,* is rare or absent in both *O. barthii* and *O. sativa*, both of which possess functional alleles of *RAE3*. Despite its importance for awn regulation, nucleotide diversity at the *RAE3* locus in *O. glaberrima* compared to *O. barthii* was at the 34th percentile of chromosome-wide levels, i.e., not remarkably low relative to other regions on chromosome 6. Highly reduced values of this ratio relative to other regions would normally be expected if the gene had experienced a hard selective sweep. However, if the *O. glaberrima* population experienced a very strong population bottleneck at roughly the same time that selection on the *RAE3* locus occurred, such that all other regions on the chromosome also showed highly reduced genetic variation (Fig. 4*D*), and if the bottleneck happened fairly recently, it would be difficult to identify a clear signature of selection. Given the inbreeding nature of cultivated African rice (23, 59, 60) and the relatively low effective population size of *O. glaberrima*, the result reported here is not inconsistent with a hard selective sweep at *RAE3*. Alternatively, other forms of selection, such as a soft sweep, may have impacted the evolutionary pathway to awnlessness in African rice. For example, resequencing data suggested that nearly half of *O. glaberrima* accessions studied, all of which carry the 48-bp deletion, have structural variation downstream of *RAE3* (Fig. 4*B* and Dataset S1); this indicates that the mutation may be found in at least two haplotypes of cultivated African rice. A soft selective sweep would be consistent with a protracted model of domestication (9, 19, 61) whereby both awned and awnless populations were cultivated under early domestication management. While awnless rice would facilitate postharvest processes, awned types offer better natural protection from birds and other seed predators under these managed scenarios, thereby balancing selective pressures on the awned and awnless phenotypes. As cultivation practices became more intensive throughout the domestication process, the 48-bp deletion would have become the favored variant, leading to virtual fixation in *O. glaberrima* with some genetic variation still retained in the surrounding region of *Ograe3* (*SI Appendix*, Fig. S13). The data are also consistent with a model of domestication for *O. glaberrima* whereby a prolonged period of domestication was punctuated by a relatively recent hard sweep favoring awnlessness conferred by the 48-bp deletion in *RAE3*.

Strong selection on multiple awn-regulating genes contributed to the domestication of *O. sativa*, while selection at a single, newly discovered locus led to awnlessness in *O. glaberrima*. In Asian cultivated rice, dysfunctional mutations in *An-1/RAE1*, *RAE2/GAD1,* and *LABA1/An-2* contributed to a reduction in awn length and morphology, whereas in African cultivated rice *RAE3* appears to be the major target of selection (Fig. 4*E*). This result is consistent with the highly stratified population structure and greater amount of genetic diversity found in both cultivated and wild forms of Asian rice. While significant subpopulation structure is detected in *O. glaberrima* (17–20), it is shallower than that observed in *O. sativa* due to the fact that *O. glaberrima* has a much narrower gene pool (Fig. 4*D*) and is more geographically restricted than *O. sativa*. The current study sheds light on convergent evolutionary processes that led to the independent domestication of Asian and African rice.

## Materials and Methods

Summary of Materials and Methods are described here, and the details are in *SI Appendix*.

### Plant Growth Conditions.

Plant materials were grown in the field of Nagoya University at Togo-cho and in the field of Kyushu University at Kasuya in Japan following the conventional agronomic calendar. The transgenic plants were grown in isolated greenhouses under long-day conditions until the 10-leaf stage, and then transferred to short-day conditions until flowering.

### Fine Mapping of RAE3 through Linkage Analysis.

For fine mapping of *OsRAE3*, we used 6,912 OGBC_4_F_3_ plants, which carry a fragment of approximately 4.5 Mb on the long arm of chromosome 6 from T65 (*O. sativa*) in the IRGC103777 (*O. glaberrima*) background. PCR for genotyping was performed with Ex-Taq polymerase (Takara Bio Inc., Kusatsu, Japan) following the manufacturer’s protocol. Markers used for genotyping of OGBC_4_F_2_ and OGBC_4_F_3_ are listed in *SI Appendix*, Table S5.

### Plasmid Construction and Generation of Transgenic Rice Plants.

To narrow down the candidate region of *RAE3*, the BAC clone OsBAC_10E15, harboring the entire 92-kb candidate region, was screened from the T65 (*O. sativa*) BAC library and shotgun-sequenced using the MiSeq platform (Illumina, San Diego, USA). The BAC clone was partially digested with *Sau*3AI, yielding fragments of approximately 10 to 30 kb that were then subcloned into the binary vector pYL-TAC7. To identify the responsible gene for *RAE3*, each CDS of four candidate genes contained in the 2-03H subclone was amplified from Nipponbare (*O. sativa*) cDNA via PCR and cloning into the pCAMBIA1380 vector. To examine the function of *OsRAE3* with or without mutation in RING-H2 domain in rice, the *Os06g0695900* CDS was cloned into pCAMBIA1300 carrying 3× FLAG on the 5′ side of the transgene (FLAG-RAE3(WT)ox). A single-point mutation in the RING-H2 domain of RAE3 (FLAG-RAE3(C136S)ox) was generated through PCR using a specific primer pair (KM154–KM155) with FLAG-RAE3(WT)ox as the template. To observe *RAE3* cell localization, the *Os06g0695900* CDS (OsRAE3(WT)) and mutated OsRAE3, OsRAE3(C136S) were cloned into pEG101 by LR cloning.

### Phenotypic Evaluation.

The three main panicles of each plant were collected for analysis. The awn lengths of the apical spikelets of the top five primary branches were measured, and this measurement was taken to represent the awn length of the whole panicle. The awned seed number was divided by the total seed number in one panicle to calculate the frequency of awned seeds per panicle.

### RAE3 Expression Data Retrieving from RED Database.

Expression values based on FPKM were retrieved from the RED [(42); http://expression.ic4r.org, searched on January 4, 2020] which is based on RNA-seq data of *O. sativa* cv. Nipponbare. The expression values corresponding to the tissues that have “normal” and “WT” without any treatment in experiment name were retrieved and calculated mean value among each tissue. We used the data from the Project IDs; DRP000391, DRP001762, SRP017256, SRP029886, SRP047482, and SRP049102.

### RNA Isolation and Quantitative Reverse-Transcription (qRT)-PCR.

For qRT-PCR analysis of target genes (*RAE1* (Os04g0350700), *RAE2* (Os08g0485500), and *RAE3* (Os06g0695900)), young panicle tissues (<1 cm in length) of several rice accessions were used. Total RNA was extracted using the RNeasy Plant Mini Kit (QIAGEN, Hilden, Germany), and first-strand cDNA synthesis was performed using the Omniscript RT Kit (QIAGEN, Hilden, Germany). The StepOne real-time PCR system (Applied Biosystems, Waltham, MA, USA) was used to analyze the relative expression levels of target genes. Expression levels of target genes were normalized to the endogenous ubiquitin transcript level, *UBQ5* (Os01g0328400). The comparative cycle threshold (△△CT) method was used to calculate relative expression levels of target genes.

### Particle Bombardment in Onion Epidermal Cell and Observation by Confocal Microscopy.

For observing transient expression of OsRAE3 in onion epidermal cells, the fusion protein of OsRAE3(WT)-YFP was constructed under the control of the 35S promoter. The strips of onion scale leaves were subjected to particle bombardment using the biolistic PDS1000/He Particle Delivery System (Bio-Rad). Bombardment was performed with a 1,100 psi rupture disc (#1652329, Bio-Rad) under the condition of 28 inch Hg (vacuum level in chamber), 1,100 psi helium pressure, and 590 MPa pressure. After bombardment with gold particles, samples were incubated at 28 °C for 16 h in the dark. The epidermal layer was peeled off, then observed under confocal laser microscope with a 40× objective (Zeiss, Oberkochen, Germany).

### Rice Protoplast Isolation and Subcellular Localization Analysis.

Rice protoplast isolation of Nipponbare seedlings and protoplast transfection were followed as described previously (62), with some modifications. Plasmid DNA was mixed with 100 µL protoplasts (about 6× 106 cells), and 150 µL PEG solution [40% (W/V) PEG 4000; Aldrich, 0.2 M mannitol, and 0.1 M CaCl2] was added and mixed by tapping. After incubation at room temperature for 10 to 20 min, 600 µL W5 solution (154 mM NaCl, 125 mM CaCl2, 5 mM KCl, and 2 mM MES at pH 5.7) was added and mixed well by inverting the tube. After centrifuge, the supernatant was removed and resuspended by adding 400 µL WI solution (0.5 M mannitol, 20 mM KCl, and 4 mM MES at pH 5.7). The solution was transferred to a 24-well plate and incubated at 22 °C in the dark for 12 to 16 h. Protoplasts were observed using a confocal laser scanning microscope (LSM700; Zeiss).

### E3 Ubiquitin Ligase Assay in Yeast.

#### Yeast Strain, Media, and Reagents.

The yeast strain used in this work is YTK2812 (MATa *leu2 trp1 his3 ade2 can1 pdr5*::*Hyg*) which is constructed in this study. Cells were grown in synthetic medium (0.69% yeast nitrogen base without amino acids, 2% D-glucose, appropriate amino acids, and nucleic acids) at 30 ˚C. To initiate degradation of IAA17 -tagged proteins, 3-IAA (cat. 19119-61, Nacalai Tesque, Kyoto, Japan) was added to the medium at 300 µM final concentration. To inhibit de novo protein synthesis, CHX (cat. 06741-04, Nacalai Tesque) was added to the medium at 200 µg/mL final concentration. For inhibition of the proteasome, MG132 (cat. 3175, Peptide Institute, Osaka, Japan) was added to the medium 20 min before CHX treatment at a final concentration of 50 µM.

#### Plasmid Construction.

To examine the function of *OsRAE3* in yeast, the *OsRAE3* CDS (*Os06g0695900*) without the transmembrane domain and expected substrate recognition site (from amino acids 54 to 166) was cloned into the p416ADH vector (63) by SpeI and BamHI. The C-terminal domain of *OsTIR1* (Os04g0395600) used in the Auxin-based degron system (51) (from amino acids 36 to 576) was amplified via PCR and introduced on the 3′ side of the *OsRAE3* fragment (FLAG-ΔRAE3(WT)-TIR) by BamHI and EcoRI. A single point mutation in the RING-H2 domain in RAE3 (FLAG-ΔRAE3(C136S)-TIR) was generated through PCR using specific primers (KM154–KM155), with FLAG-ΔRAE3(WT)-TIR as the template. The plasmid which is FLAG-ΔRAE3(WT)-TIR was named pOK832 and FLAG-ΔRAE3(C136S)-TIR was named pOK833. AtIAA17 (At1g04250) was cloned using the pOK521 plasmid (64) as a template and added the 3×HA tag to the 5′ side to produce 3× HA-IAA17-p415ADH construct used as a substrate for FLAG-ΔRAE3-TIR. The constructs for observing cell localization of chimeric OsRAE3 protein fused with GFP were made by using NEBuilder as following manufacture protocol. GFP sequence was amplified with specific primers (KBU71-KBU72) using pMDC111 as a template. PCR products of GFP fused with 19 to 20 bp of complementary sequence of vector plasmid were transferred into pOK832 or pOK833 after EcoRI treatment using the NEBuilder Hifi assembly kit (New England BioLabs, Ipswich, MA, USA) with 50 °C for 20 min.

#### Diversity Analysis of RAE3.

Individual resequencing datasets were downloaded from the internet as raw reads and aligned to the Nipponbare reference genome using BWA software (65, 66) for alignment, and GATK’s HaplotypeCaller algorithm (67–70) for variant-calling. A uniform set of parameters was used to ensure data quality and to enable integration of datasets. Resulting data were utilized for examining the frequency of the 48-bp deletion in wild and cultivated African rice (n = 62 *O. barthii* and 120 *O. glaberimma* for which the 48-bp deletion were called), constructing haplotypes across the *RAE3* genomic region in both African and Asian rice (n = 23 *O. barthii*, 100 *O. glaberrima*, and 100 *O. sativa*), and analyzing nucleotide diversity across chromosome 6 (n = 93 *O. barthii*, 134 *O. glaberrima*, 110 *O. sativa*, and 41 *O. rufipogon*). For haplotype construction, accessions with greater than 10% missing calls were filtered out as were SNPs with greater than 0.05% missingness.

## Supplementary Material

Appendix 01 (PDF)

Dataset S01 (CSV)

## Data Availability

All study data are included in the article and/or *SI Appendix*.
