# Supplementary material for: Regulator of Awn Elongation 3, an E3 ubiquitin ligase, is responsible for loss of awns during African rice domestication
Source: Proc Natl Acad Sci U S A. 2023 Jan 17;120(4):e2207105120. doi: 10.1073/pnas.2207105120 (PMC9942864; doi:10.1073/pnas.2207105120)
Supplement: Supplementary file 1 — Appendix 01 (PDF) [file pnas.2207105120.sapp.pdf]

PNAS

Supporting Information for

REGULATOR OF AWN ELONGATION 3, an E3 ubiquitin ligase, is responsible for loss of awns during African rice domestication

Kanako Bessho-Uehara[†], Kengo Masuda[†], Diane R. Wang[†], Rosalyn B. Angeles-Shim, Keisuke Obara, Keisuke Nagai, Riri Murase, Shin-ichiro Aoki, Tomoyuki Furuta, Kotaro Miura, Jianzhong Wu, Yoshiyuki Yamagata, Hideshi Yasui, Michael Kantar, Atsushi Yoshimura, Takumi Kamura, Susan R. McCouch[*], Motoyuki Ashikari[*]

[*]Corresponding authors:

Motoyuki Ashikari <ashi@agr.nagoya-u.ac.jp>, Susan McCouch <srm4@cornell.edu>

This PDF file includes:

- Supporting information text
- Figures S1 to S13
- Tables S1 to S5
- SI References

Other supporting materials for this manuscript include the following:

- Datasets S1

Supporting Information Text

Materials and Methods

Plant materials and growth conditions. Plant materials were grown in the field of Nagoya University at Togo-cho, Aichi and in the field of Kyushu University at Kasuya, Fukuoka, Japan following the conventional agronomic calendar. $OGBC_4F_1$ was a backcrossed inbred line (BIL) that was crossed with IRGC103777 (*O. glaberrima*) four times, with F_1 derived from the cross between IRGC103777 (*O. glaberrima*) and Taichung 65 (T65) (*O. sativa japonica*) at Kyushu University. $OGBC_4F_2$ and $OGBC_4F_3$ were derived by self-crossing $OGBC_4F_1$ and $OGBC_4F_2$, respectively. GLSLs which are the chromosome segment substitution lines carrying small segments of IRGC104038 (*O. glaberrima*) chromosomes in *O. sativa* (cv. Koshihikari) genetic background were provided from the Honda Research Institute (Kisarazu, Japan) (1). The transgenic plants were grown in isolated greenhouses under long-day conditions until the 10-leaf stage, and then transferred to short-day conditions until flowering. For the *RAE3* complementation test and overexpression analyses, SGR19 showing the awnless phenotype was used as the transformation background. SGR19 is a BIL carrying chromosome segment substitutions of WK18 (*O. glaberrima*) on chromosomes 6 and 8, which harbor *Ograe3* and *OgRAE2*, respectively, in the T65 genetic background (**Fig. S3A**). Information about the accessions used for the diversity and selective sweep analyses is provided in **Table S3**.

Fine mapping of *RAE3* through linkage analysis. For linkage analysis of *OsRAE3*, we used 196 $OGBC_4F_2$ plants. For fine mapping of *OsRAE3*, we used 6,912 $OGBC_4F_3$ plants derived from the self-fertilization of $OGBC_4F_2$, which carries a fragment of

approximately 4.5 Mb on the long arm of chromosome 6 from T65 (*O. sativa*) in the IRGC103777 (*O. glaberrima*) background. PCR for genotyping was performed with Ex-Taq polymerase (Takara Bio Inc., Kusatsu, Japan) following the manufacturer's protocol. Markers used for genotyping of $OGBC_4F_2$ and $OGBC_4F_3$ are listed in **Table S5**.

Plasmid construction and generation of transgenic rice plants. To narrow down the candidate region of *RAE3*, the BAC clone OsBAC_10E15, harboring the entire 92-kb candidate region, was screened from the T65 (*O. sativa*) BAC library and shotgun-sequenced using the MiSeq platform (Illumina, San Diego, USA). The BAC sequence was assembled using the Genetyx software package (Genetyx Co., Tokyo, Japan). The annotated genes were compared to gene annotations in RAP-DB (http://rapdb.dna.affrc.go.jp/viewer/gbrowse/build4). The BAC clone was partially digested with *Sau*3AI, yielding fragments of approximately 10–30 kb that were then sub-cloned into the binary vector pYL-TAC7. Six sub-clones covering the entire 92-kb candidate region (**Fig. 2c**) were selected and used for complementation test. For screening of the BAC and sub-clones, we used Ex-Taq polymerase (Takara Bio Inc., Kusatsu, Japan) under the following conditions: 95°C for 5 min; 35 cycles of 95°C for 30 s, 55°C for 30 s, and 72°C for 30 s; and a final extension at 72°C for 7 min. Only the sub-clone designated 2-03H could complement the awned phenotype (5 positive transgenic lines out of 6 in total transgenic calli, and all the lines showed awned phenotype) (**Fig. 2d**). To identify the responsible gene for *RAE3*, the CDSs of four candidate genes contained in the 2-03H sub-clone were amplified from Nipponbare (*O. sativa*) cDNA via PCR using Prime Star GXL (Takara Bio Inc., Kusatsu, Japan) for cloning into the

pBluescript vector. DNA fragments containing each gene were transferred into the pCAMBIA1380 vector carrying the ubiquitin promoter upstream of the multiple-cloning site. Only the line carrying the Os06g0695900 construct could complement the awned phenotype (8 positive transgenic lines out of 12 in total transgenic calli) (**Fig. 2e**). Six lines showed awned phenotype and the quantitative data are found in **Fig. S3** and **Table S1**. To examine the function of *OsRAE3* with or without mutation in RING-H2 domain in rice, the *Os06g0695900* CDS was cloned into pCAMBIA1300 carrying 3× FLAG on the 5′ side of the transgene (FLAG-RAE3(WT)ox). A single point mutation in the RING-H2 domain of RAE3 (FLAG-RAE3(C136S)ox) was generated through PCR using a specific primer pair (KM154–KM155) with FLAG-RAE3(WT)ox as the template. The constructs were subjected to the standard protocol of *Agrobacterium* (strain EHA105)-mediated rice transformation. Transgenic lines were selected on Murashige & Skoog (MS) medium containing 50 mg hygromycin (Sigma, St. Louis, MO, USA). Around 70% of transgenic lines have insertion with either construct (OsRAE3(WT): 11/15, OsRAE3(C136S): 7/10) but only lines with OsRAE3(WT)ox showed the awned phenotype. Quantitative data of awn phenotypes (awned seed ratio per panicle and awn length) of 3 positive transgenic lines are found in **Table S1**. To observe *RAE3* cell localization, the *Os06g0695900* CDS (OsRAE3(WT)) and mutated OsRAE3, OsRAE3(C136S) was cloned into the pENTR/SD/D-topo vector (Invitrogen, Waltham, MA, USA). OsRAE3(C136S)/pENTR was generated through site directed mutagenesis PCR using a specific primer pair (KM154–KM155) with OsRAE3(WT)/pENTR as the template. Then genes cloned into pENTR were transferred into pEG101 by using Gateway LR clonase II (ThermoFisher

Scientific, MA, USA) by following manufactured protocol. The constructs in pEG101 were used for transient expression analysis in onion epidermal cells and rice protoplast.

Primers. The primers used in this study are listed in **Table S5**.

Phenotypic evaluation. Panicles of the parental plants (WK18 (*O. glaberrima*), T65 (*O. sativa japonica*), and $OGBC_4$ lines), CSSLs and transgenic plants carrying BAC sub-clones, and *RAE3* overexpression lines were harvested after seed maturation. Panicles were sampled from 10 plants for measurement of awn length and the frequency of awned seeds per panicle. The three main panicles of each plant were collected for analysis. The awn lengths of the apical spikelets of the top five primary branches were measured, and this measurement was taken to represent the awn length of the whole panicle. The awned seed number was divided by the total seed number in one panicle to calculate the frequency of awned seeds per panicle.

Visualization of local genome rearrangement around the site of *RAE3*: comparison among *O. sativa, O. glaberrima*, and *O. barthii*. To visualize differences in genome structure around the putative location of *RAE3*, the genome sequences of *O. sativa*, *O. glaberrima*, and *O. barthii* were compared. A portion of the *O. sativa* genome sequence containing the candidate *RAE3* locus was obtained by subsetting 28,983,088–29,153,151 bp of chromosome 6 from the *O. sativa* Nipponbare genome sequence, IRGSP-1.0, distributed via the RAP-DB (https://rapdb.dna.affrc.go.jp/) (2, 3). The *O. glaberrima* genomic region corresponding to the *O. sativa RAE3* location was roughly identified

based on Basic Local Alignment Search Tool (BLAST) hits of the first and last 100 bp of the *O. sativa* subset sequence. The nucleotide sequence of the identified genomic region, which was 21,660,059–21,794,912 bp on chromosome 6, was obtained from the genome sequence labeled Oryza_glaberrima_V1 on Ensembl Plants (https://plants.ensembl.org) (4). To obtain the corresponding sequence of *O. barthii*, we conducted *de novo* assembly of two BAC clone sequences (OBART0063E22 and OBART0027J20) that were screened via PCR amplification of sequences around the *RAE3* locus and provided by the National Agriculture and Food Research Organization of Japan. A sequencing library for the two BAC clones was prepared using the NEBNext® Ultra™ DNA Library Prep Kit for Illumina® (New England BioLabs, Ipswich, MA, USA) and then subjected to sequencing using the next-generation sequencing platform MiSeq (Illumina, San Diego, USA) with MiSeq Reagent Kit v3 (150-cycle) (Illumina, San Diego, USA). The 630,277 reads obtained for the BAC clones were first aligned to the *E. coli* genome (GenBank: U00096.3) and pIndigoBAC-5 (GenBank: EU140754.1), which is the BAC's backbone, using the BWA-MEM algorithm (5). Unmapped paired reads were extracted using SAMtools with the option -f 13 for the view function (6). The unmapped reads were then assembled using ABySS (7) with the setting k = 51, which provided the best result in the tested k values of 31–71. Assembled contigs longer than 5 kb were applied to contig extension using the PRICE tool (8). Parameters for PriceTI were set as follows: -fs ./path/to/reads.fastq 200 -icf ./path/to/contigs.fasta 1 1 5 -nc 10 -target 90 0 -o ./path/to/output.fa. The resultant contigs were further processed using SSPACE-standard for scaffolding (9), with parameter -x set to 1. The longest scaffold, containing 6 Ns in a gap, was confirmed to correspond to the *O. sativa* subset sequence. BLAST

comparison of the obtained sequences was performed using WebACT to create the comparison files required by Artemis Comparison Tools (ACT) (10, 11). The local genome sequence of the putative *RAE3* location was then visualized using ACT, which was tuned to eliminate line segments connecting sequences of relatively low similarity.

***RAE3* Expression data retrieving from RED database.** Expression values based on FPKM were retrieved from the Rice Expression Database (RED (12); http://expression.ic4r.org, searched on Jan 4, 2020) which is based on RNA-seq data of *O. sativa* cv. Nipponbare. The expression values corresponding to the tissues that have "normal" and "WT" without any treatment in experiment name were retrieved and calculated mean value among each tissue. We used the data from the Project IDs; DRP000391, DRP001762, SRP017256, SRP029886, SRP047482, and SRP049102.

RNA isolation and quantitative reverse-transcription (qRT)-PCR. For qRT-PCR analysis of target genes (*RAE1* (Os04g0350700), *RAE2* (Os08g0485500) and *RAE3* (Os06g0695900)), young panicle tissues (< 1 cm in length) of Koshihikari (*O. sativa japonica*), GLSL14, GLSL20 and GLSL26 (in which segments of chromosomes 4, 6, and 8 were individually substituted into *O. glaberrima* from the Koshihikari genetic background, as indicated in **Fig. 1**) were used as samples. Total RNA was extracted using the RNeasy Plant Mini Kit (QIAGEN, Hilden, Germany), and first-strand cDNA synthesis was performed using the Omniscript RT Kit (QIAGEN, Hilden, Germany). The StepOne Real-Time PCR system (Applied Biosystems, Waltham, MA, USA) was used to analyze the relative expression levels of target genes. Expression levels of target genes

were normalized to the endogenous ubiquitin transcript level (*Os01g0328400*). The comparative cycle threshold (△△CT) method was used to calculate relative expression levels of target genes. Primers used in this study are listed in **Table S5**.

Alignment of the *RAE3* sequence and *RAE3* phylogenetic tree. After visualizing the genome rearrangement around the *RAE3* location, the CDS sequence of *Os06g0695900,* which corresponds to *OsRAE3*, and the 3-kb upstream and 1-kb downstream sequences, were retrieved from the *O. sativa* genome as the promoter and terminator regions. Corresponding sequences of the promoter, CDS and terminator regions of *RAE3* were retrieved manually from the *O. glaberrima* and *O. barthii* genomes. The *RAE3* sequences of *O. sativa*, *O. glaberrima* and *O. barthii* were assembled using Genetyx software (Genetyx Co., Tokyo, Japan). Amino acid sequences of *RAE3* orthologues from various plant species were identified through reciprocal best-match BLAST searches using the Phytozome v12.1 (https://phytozome-next.jgi.doe.gov) and National Center for Biotechnology Information (NCBI; https://www.ncbi.nlm.nih.gov) databases. Accession numbers and locus identifications were derived from Phytozome v12.1. Amino acid sequences were aligned using the ClustalW program with default settings. The number of amino acid substitutions between each pair of *RAE3* orthologues was estimated using the Jones-Taylor-Thornton (JTT) model with the complete-deletion option. To construct the phylogenetic tree, the Neighbor-Joining method was applied in Geneious software (version 9.0). Bootstrap values from 100 replications are shown at branch nodes (values of 50% or greater are shown).

Scanning electron microscopy. The young panicles of GLSL26 was fixed in starch-based glue for microscopic observation. The samples were viewed using the SEM (S-3000N, Hitachi, Tokyo, Japan) scanning electron microscope which was set at -5°C inside temperature and at 3.2 kV.

Particle bombardment in onion epidermal cell and observation by confocal microscopy. For observing transient expression of OsRAE3 in onion epidermal cells, the fusion protein of OsRAE3(WT)-YFP was constructed under the control of the 35S promoter. Three mg of gold particles (1 µm, Bio-Rad micro carrier, Bio-Rad, Hercules, USA) was mixed with 1ml of 99% ethanol (Wako, Japan) and vortex for 2 min. Spin the sample at 10,000 g in a microcentrifuge for 1 min and remove the supernatant. Repeat washing by ethanol 3 times. Add 50 µl 50% (w/v) glycerol to washed gold particles and mixed it well. Add 5 µg of OsRAE3(WT)-YFP plasmid, 50 µl of 2.5 M $CaCl_2$, 20 µl of 0.1 M spermidine (Wako, Japan) to 50 µl of gold particles and mess up to 125 µl by distilled water. After adding all the solution, vortex thoroughly for 3 min. Spin the sample at 10,000 g in a microcentrifuge for 1 min and remove the supernatant. Add 250 µl of 99% ethanol and vortex vigorously. Spin the sample at 10,000 g in a microcentrifuge for 1 min and remove the supernatant. Add 50 µl of 99% ethanol and vortex vigorously. Onion bulbs (*Allium cepa*) were purchased from the supermarket (Nagoya, Japan). The onion was kept at room temperature before being used for particle bombardment. The onion bulb scale leaves were cut into strips of approximately 2 cm × 3 cm, and a strip of scale leaf was placed on a stack of wet Kimwipes in a 9 cm Petri dish. Then, the strips of onion scale leaves were subjected to particle bombardment using the

biolistic PDS1000/He Particle Delivery System (Bio-Rad). Bombardment was performed with a 1100 psi rupture disc (#1652329, Bio-Rad) under the condition of 28inchHg (vacuum level in chamber), 1100 psi helium pressure, 590 MPa pressure. After bombardment with gold particles, samples were incubated at 28ºC for 16 h in the dark. The epidermal layer was peeled off, soaked in liquid MS medium with or without 2 µM FM4-64 for 15 minutes, covered with foil, and mounted on glass slides for observation of YFP fluorescence using an LSM510 inverted confocal laser microscope with a 40× objective (Zeiss, Oberkochen, Germany). Excitation wavelengths of 514 and 488 nm, and detection wavelengths of 595–620 and > 640 nm, were used for detection of YFP and FM4-64, respectively.

Rice protoplast isolation and subcellular localization analysis. Rice protoplast isolation of Nipponbare seedlings and protoplast transfection was followed as described previously (13), with some modifications. Plasmid DNA were mixed with 100 µL protoplasts (about 6× 106 cells). 150 µL PEG solution [40% (W/V) PEG 4000; Aldrich, 0.2 M mannitol and 0.1 M CaCl2] were added and mixed by tapping. The solution was incubated at room temperature for 10-20 min in the dark. After incubation, 600 µL W5 solution (154 mM NaCl, 125 mM CaCl2, 5 mM KCl and 2 mM MES at pH 5.7) were added and mixed well by gently inverting the tube. After centrifugation at 500g for 5 minutes at room temperature, remove the supernatant and resuspend by adding 400µL of WI solution (0.5 M mannitol, 20 mM KCl and 4 mM MES at pH 5.7). The solution was transferred to a 24-well plate and incubated at 22°C in the dark for 12-16h. Protoplasts were observed using a confocal laser scanning microscope (LSM700; Zeiss).

E3 ubiquitin ligase assay in yeast

Yeast strain, media, and reagents. The yeast strain used in this work is YTK2812 (MATa *leu2 trp1 his3 ade2 can1 pdr5*::*Hyg*) which is constructed in this study. Cells were grown in synthetic medium (0.69% yeast nitrogen base without amino acids, 2% D-glucose, appropriate amino acids and nucleic acids) at 30˚C. To initiate degradation of IAA17 -tagged proteins, 3-indoleacetic acid (IAA) (cat. 19119-61, Nacalai Tesque, Kyoto, Japan) was added to the medium at a final concentration of 300 μM. To inhibit *de novo* protein synthesis, cycloheximide (CHX) (cat. 06741-04, Nacalai Tesque) was added to the medium at a final concentration of 200 μg/mL. For inhibition of the proteasome, MG132 (cat. 3175, Peptide Institute, Osaka, Japan) was added to the medium 20 min before CHX treatment at a final concentration of 50 μM.

Plasmid construction. To examine the function of *OsRAE3* in yeast, the *OsRAE3* CDS (Os06g0695900) without the transmembrane domain and expected substrate recognition site (from amino acids 54–166) was cloned into the p416ADH vector (14) by SpeI and BamHI. The C-terminal domain of *OsTIR1* (Os04g0395600) used in the Auxin-based degron system (15) (from amino acids 36–576) was amplified via PCR and introduced on the 3′ side of the *OsRAE3* fragment (FLAG-ΔRAE3(WT)-TIR) by BamHI and EcoRI. A single point mutation in the RING-H2 domain in RAE3 (FLAG-ΔRAE3(C136S)-TIR) was generated through PCR using specific primers (KM154–KM155), with FLAG-Δ RAE3(WT)-TIR as the template. The plasmid which is FLAG-ΔRAE3(WT)-TIR was named pOK832 and FLAG-ΔRAE3(C136S)-TIR was named pOK833. AtIAA17

(At1g04250) was cloned using the pOK521 plasmid (16) as a template and added the 3xHA tag to the 5′ side to produce 3× HA-IAA17-p415ADH construct used as a substrate for FLAG- Δ RAE3-TIR. The constructs for observing cell localization of chimeric OsRAE3 protein fused with GFP were made by using NEBuilder as following manufacture protocol. GFP sequence was amplified with specific primers (KBU71-KBU72) using pMDC111 as a template. PCR products of GFP fused with 19 to 20 bp of complementary sequence of vector plasmid were transferred into pOK832 or pOK833 after EcoRI treatment using the NEBuilder Hifi assembly kit (New England BioLabs, Ipswich, MA, USA) with 50 degrees C for 20 min.

Immunoblot analysis. Total cell lysates were prepared through the alkaline-trichloroacetic acid method. Proteins were separated by SDS-PAGE and transferred to an Immobilon polyvinylidene difluoride membrane (Millipore, Billerica, MA, USA). The membrane was incubated with anti-HA (TANA2; Medical & Biological Laboratories, Nagoya, Japan), anti-DDDDK (FLA-1; Medical & Biological Laboratories, Nagoya, Japan), or anti-Pgk1 (22C5D8; ThermoFisher Scientific, MA, USA) antibody. HRP-conjugated anti-mouse IgG (Sigma, MO, USA) was used as the secondary antibody. Immunodetection was performed using the Chemi-Lumi One L system (Nacalai Tesque, Kyoto, Japan) with a bioanalyzer (LAS4000 mini; GE Healthcare Biosciences, Piscataway, NJ) or X-ray films.

Subcellular localization of chimeric RAE3 protein in yeast. ΔRAE3(WT)-TIR1-GFP, ΔRAE3(C136S)-TIR1-GFP constructs were transformed into YTK2812 strain by

chemical transformation. The p416-ADH-FLAG plasmid was used as negative control. The transgenic yeast cells were grown to log phase in synthetic medium lacking uracil, and observed under a fluorescence microscope (AxioObserver Z1; Carl Zeiss, Oberkochen, Germany) equipped with a CMOS camera (ORCA-fusion; Hamamatsu Photonics, Hamamatsu, Japan). Images were obtained using ZEN3.3 pro software (Carl Zeiss) with appropriate condition and processed using Photoshop CS3 (Adobe, San Jose, USA).

Diversity Analysis of *RAE3*. Re-sequencing data from chromosome 6 were obtained from Wang et al., 2014 (17), 3KGenomes Project, 2014 (18), Melandri et al., 2021 (19), Duitama et al., 2015 (20), Xu et al., 2012 (21), and Meyer et al., 2016 (22) (corresponding to GenBank accession IDs; PRJNA13765, PRJNA30379, PRJEB6180, PRJNA202926, PRJNA657887, SRP023273, SRA023116, SRP071857). Individual re-sequencing datasets were downloaded from the internet as raw reads and aligned to the Nipponbare reference genome using BWA software (23, 24) for alignment, and GATK's HaplotypeCaller algorithm (25–28) for variant-calling. A uniform set of parameters was used to ensure data quality and to enable integration of datasets. Resulting data were utilized for examining the frequency of the 48-bp deletion in wild and cultivated African rice (n = 62 *O. barthii* and 120 *O. glaberimma* for which the 48-bp deletion were called), constructing haplotypes across the *RAE3* genomic region in both African and Asian rice (n = 23 *O. barthii*, 100 *O. glaberrima*, and 100 *O. sativa*), and analyzing nucleotide diversity across chromosome 6 (n = 93 *O. barthii*, 134 *O. glaberrima*, 110 *O. sativa*, and 41 *O. rufipogon*). For haplotype construction, accessions with greater than 10% missing

calls were filtered out as were SNPs with greater than 0.05% missingness. Nucleotide diversity was computed in 10-kb bins for all 10-kb windows with a bin-average SNP missing data rate < 20%. *RAE3* nucleotide diversity ratio ($\pi_{glaberrima}/\ \pi_{barthii}$) was computed using 50-SNP sliding windows with a 2-SNP step size.

PCR amplification with the specific primers around 48-bp deletion of *RAE3*. One-cm of leaf blades were sampled from several accessions of *O. barthii* and *O. glaberrima* (accession numbers are listed in **Table S4**) and extracted genomic DNA using ethanol precipitation method. Genomic DNA of Koshihikari (*O. sativa*), WK18 (*O. glaberrima*) and the plasmid OsRAE3(WT)/pCAMBIA1380 were used as positive control. For PCR amplification, we used Prime Star polymerase (Takara Bio Inc., Kusatsu, Japan) under the following conditions: 95°C for 5 min; 30 cycles of 95°C for 30 s, 55°C for 30 s, and 72°C for 30 s; and a final extension at 72°C for 5 min. Then PCR amplicons were viewed under gel electrophoresis by running a 1% agarose/TAE gel for 15 min at 100V.

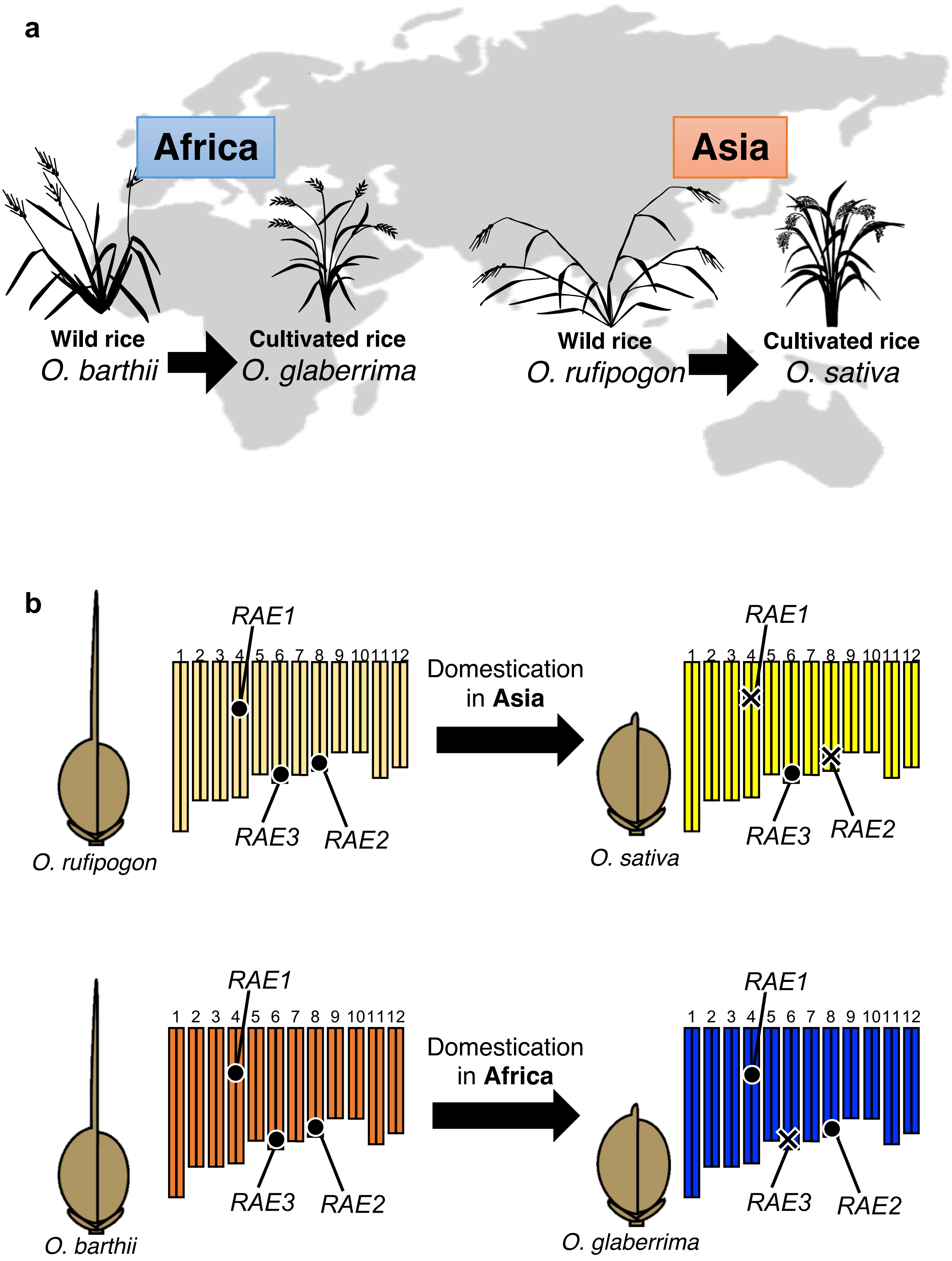

Figure S1. Independent rice domestication occurred in Asia and Africa. (a) Asian cultivated rice, *O. sativa,* was derived from the wild rice species *O. rufipogon*. African cultivated rice, *O. glaberrima,* was derived from the wild rice species *O. barthii*. (b) Gene selection model according to awnless phenotype during rice domestication in Asia (upper) and Africa (lower).

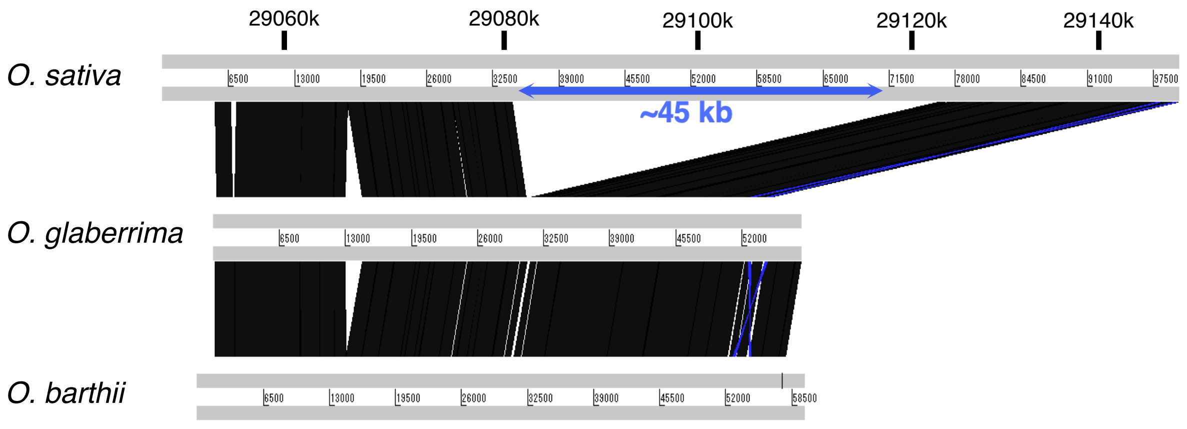

Figure S2. Chromosome synteny around the *RAE3* candidate region of chromosome 6: comparison among *O. sativa*, *O. glaberrima* and *O. barthii*. Black lines indicate syntenic regions and blue lines indicate inversion between pairs of species. The numbers between gray lines indicate the physical position (bp) of query sequence. The physical position (kb) of chromosome 6 of *O. sativa* cv. Nipponbare was exhibited top of the figure.

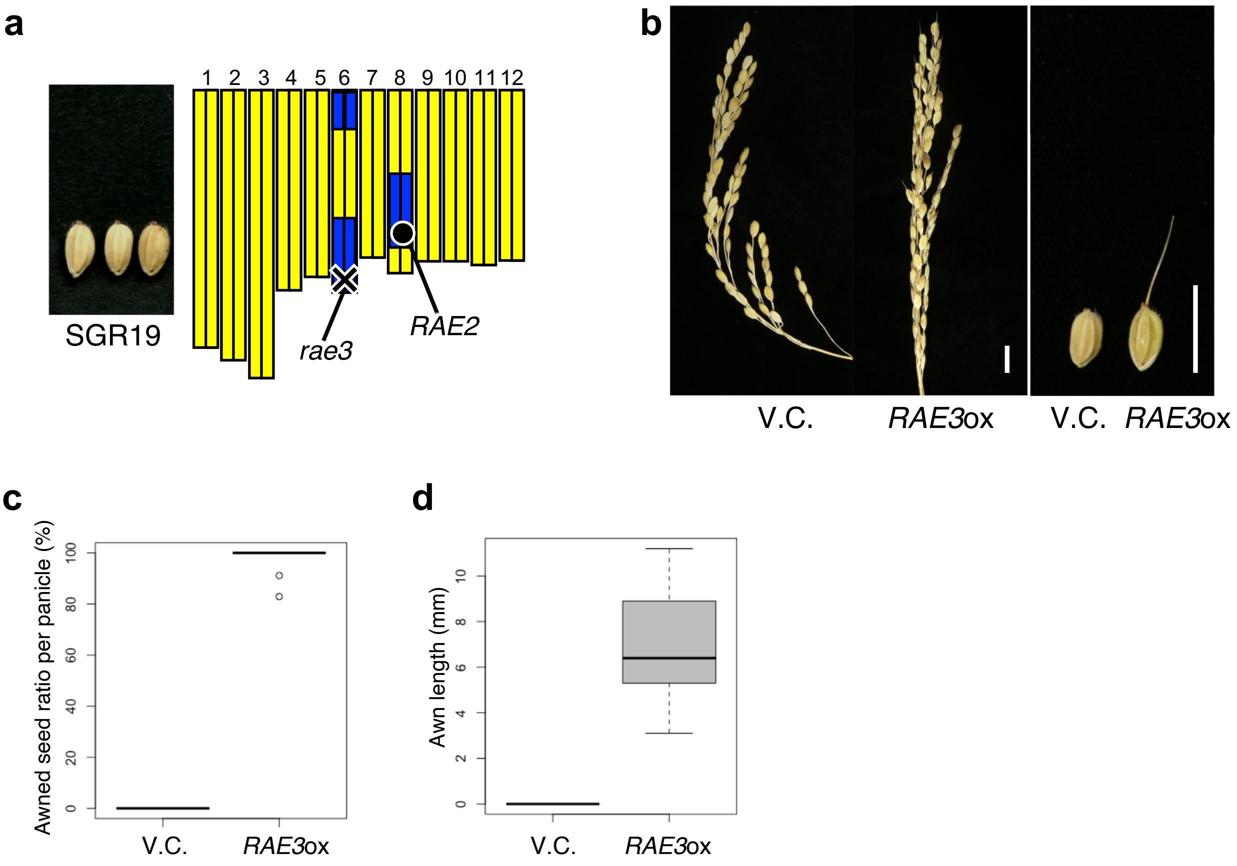

Figure S3. Quantitative data on awn phenotype in transgenic plants of *RAE3*. (a) Seed phenotypes of SGR19 carrying an *O. glaberrima* chromosome segments (indicated by blue) harboring *RAE2* (chromosome 8) and *RAE3* (chromosome 6) in *O. sativa* cv. T65 background (indicated by yellow). (b–d) Evaluation of transgenic plants with the plasmid vector pGWB501 (vector control (V.C.)) and *RAE3* gene (pUBQ::RAE3, RAE3ox). Panicle and seed phenotype (b), frequency of awned seeds per panicle (c), and awn length (d). No visible awn was observed in V.C.. Scale bar represents 1 cm.

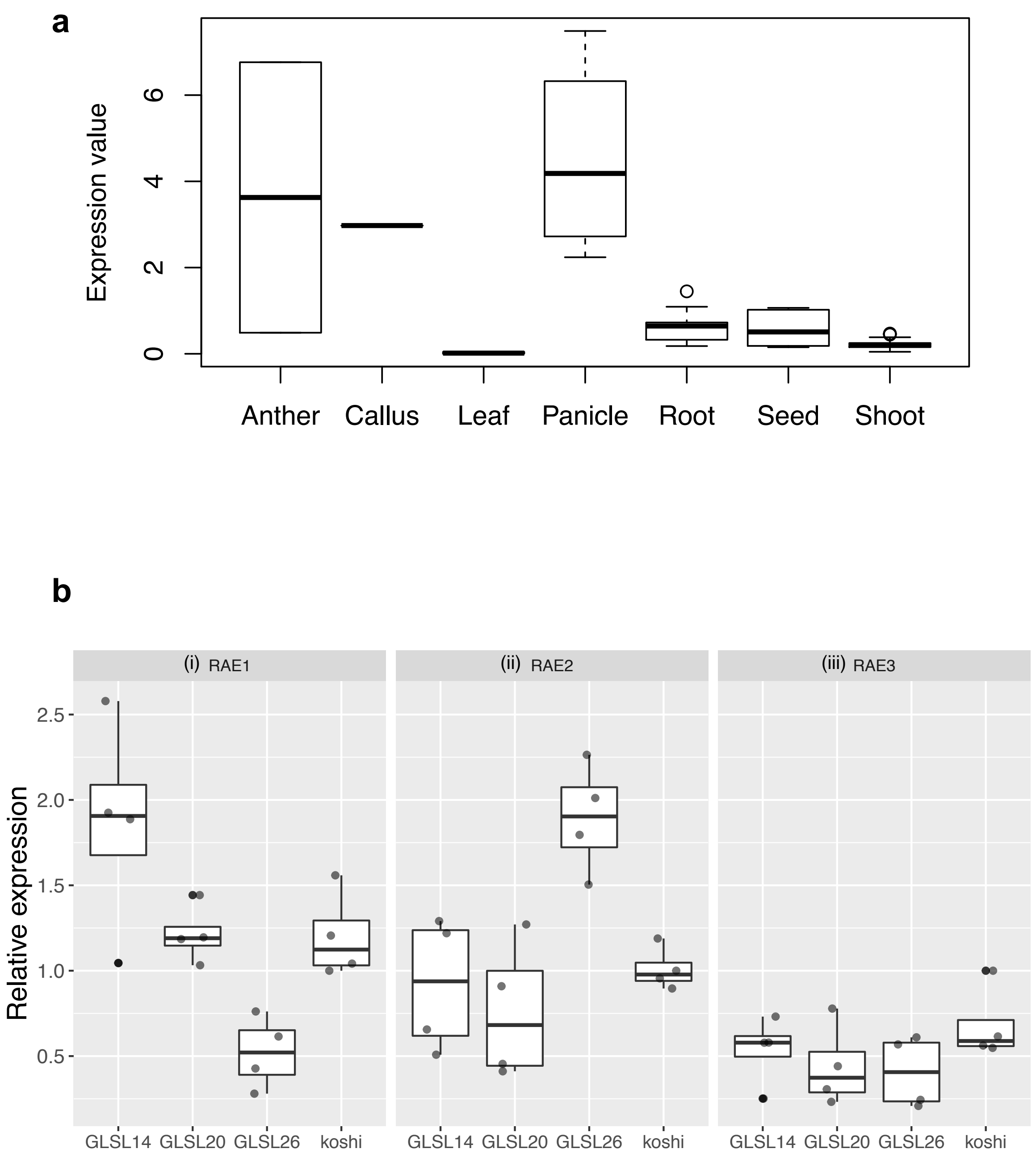

Figure S4. Expression pattern of *RAE* genes. (a) *OsRAE3* expression pattern in several organs of Nipponbare (*O. sativa*). Data from Rice Expression Database (http://expression.ic4r.org). (b) Relative expression levels of (i) *RAE1*, (ii) *RAE2* and (iii) *RAE3* genes in several CSSLs and Koshihikari (*O. sativa*) done by this study. Genotype and the alleles of 3 genes of all 4 lines are described in Fig 1.

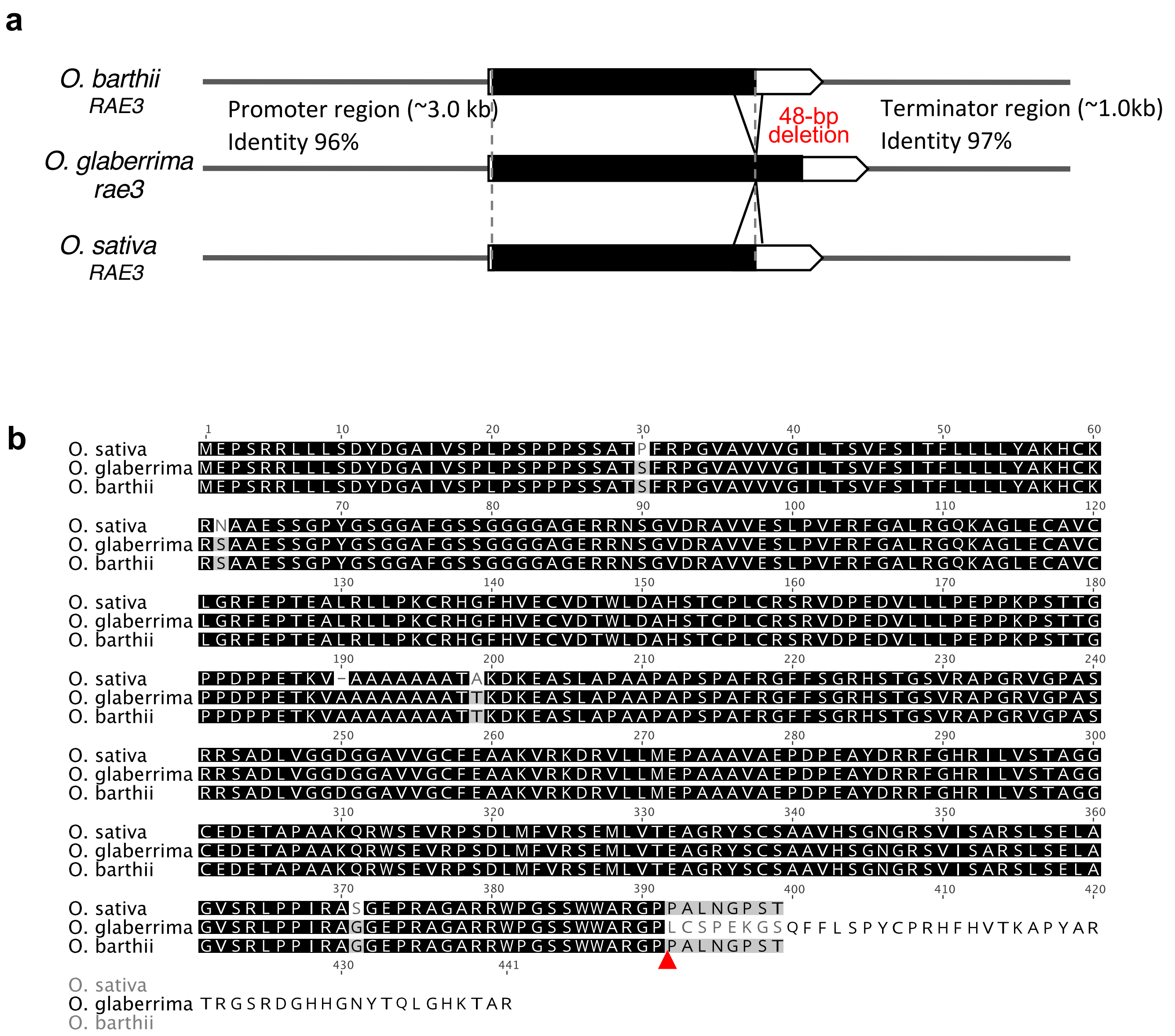

Figure S5. Typical haplotypes of functional *RAE3* and dysfunctional *rae3*. (a) Graphical comparison of the *RAE3* open reading frame (ORF) region. The white rectangles indicate untranslated regions, and black rectangles indicate exons. *RAE3* has no introns. SNPs are present in the promoter region and terminator region between *O. barthii* and *O. glaberrima*. (b) Amino acid sequence alignment among *O. barthii, O. glaberrima* and *O. sativa*. Red rectangle indicates the position of 48-bp deletion in *O. glaberrima*.

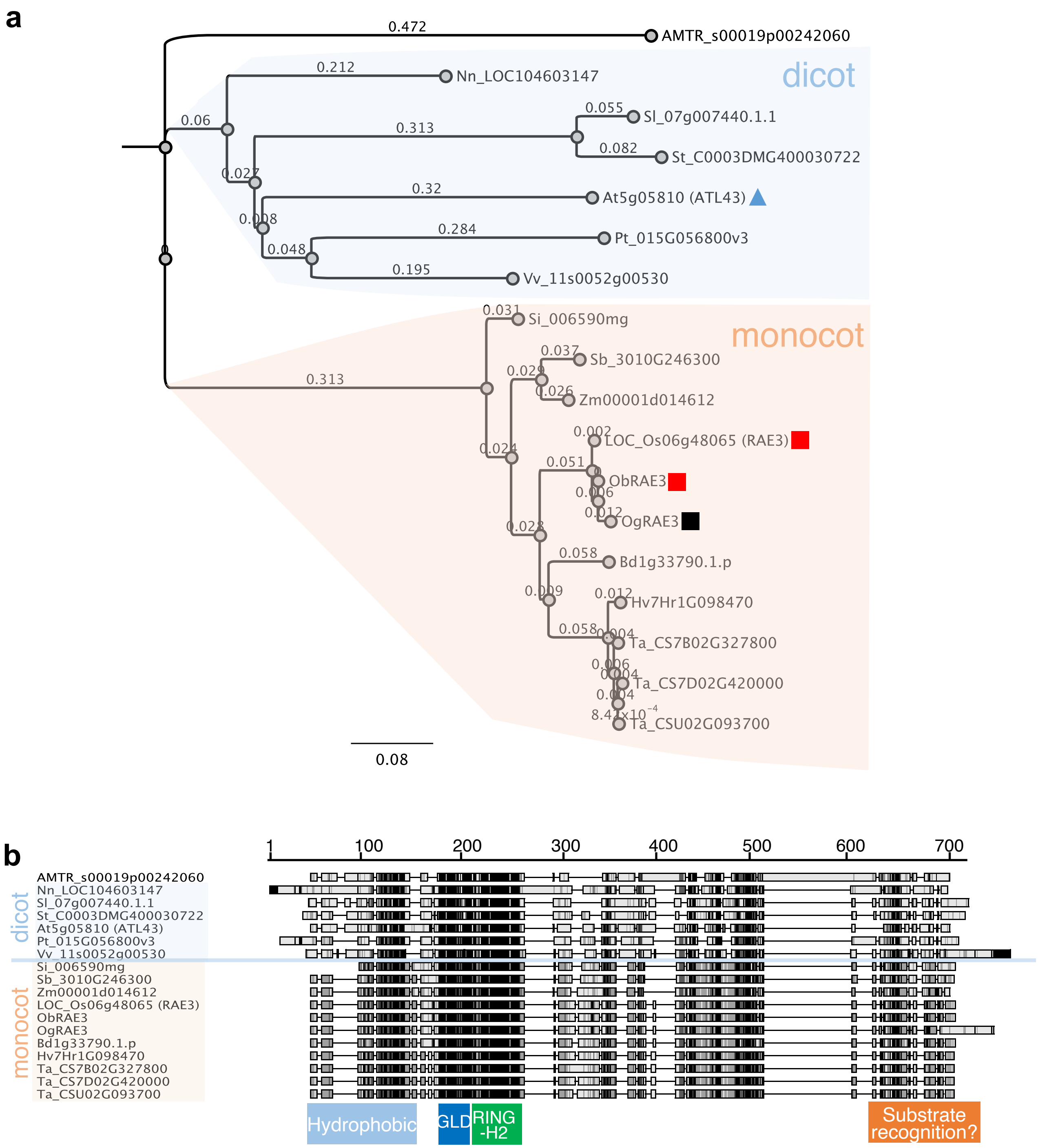

Figure S6. Phylogenetic relationship of *RAE3*. (a) Phylogenic tree of *RAE3* and its homologues in various plant species. Red and black squares indicate functional and dysfunctional *RAE3* respectively. Blue triangle indicates *RAE3* orthologue in Arabidopsis, *ATL43*. Blue and orange shade indicate dicot and monocot clade respectively. (b) Protein alignment of *RAE3* orthologues of various plant species. Similarity was indicated by color; Black, identical; Gray, similar; Pale gray, different. Thin lines indicate no alignment with the regions among species. The numbers above indicate amino acid position. Os, *Oryza sativa*; Ob, *Oryza barthii*; Og, *Oryza glaberrima*; Si, Setaria italica; Sb, *Sorghum bicolor*; Zm, Zea mays; Bd, *Brachypodium distachyon*; Hv, Hordeum vulgare; Ta, *Triticum aestivum*; Amtr, *Amborella trichopoda*; Nn, Nelumbo nucifera; Sl, *Solanum lycopersicum*; St, *Solanum tuberosum*; At, *Arabidopsis thaliana*; Pt, *Populus trichocarpa*; Vv, *Vitis vinifera*.

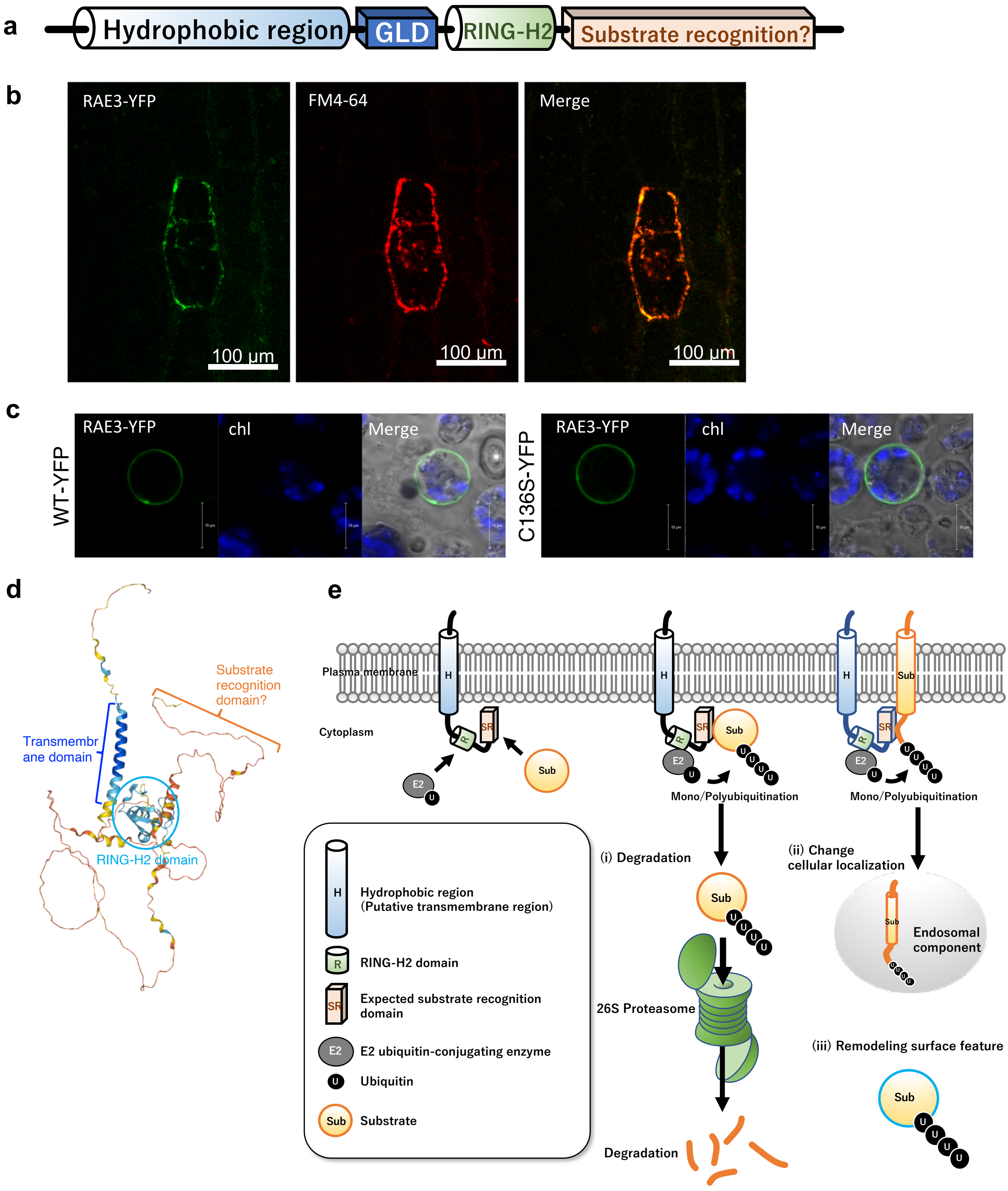

Figure S7. Structure of *RAE3* and its cellular localization. (a) Schematic diagram of RAE3. The C-terminal region is probable substrate recognition domain, so with question mark. (b) Cellular localization of RAE3 protein in onion epidermal cell. (c) Cell localization of OsRAE3(WT) and OsRAE3(C136S) protein in rice protoplast. Scale bars represent 10 μm. (d) The 3D structure model of OsRAE3 predicted by AlphaFold2 retrieved from Uniprot database. (e) RAE3 function model *in vivo*. Potentially ubiquitination contributes to (i) substrate degradation via 26S proteasome, (ii) change cellular localization of substrate, and (iii) remodel the protein surface feature.

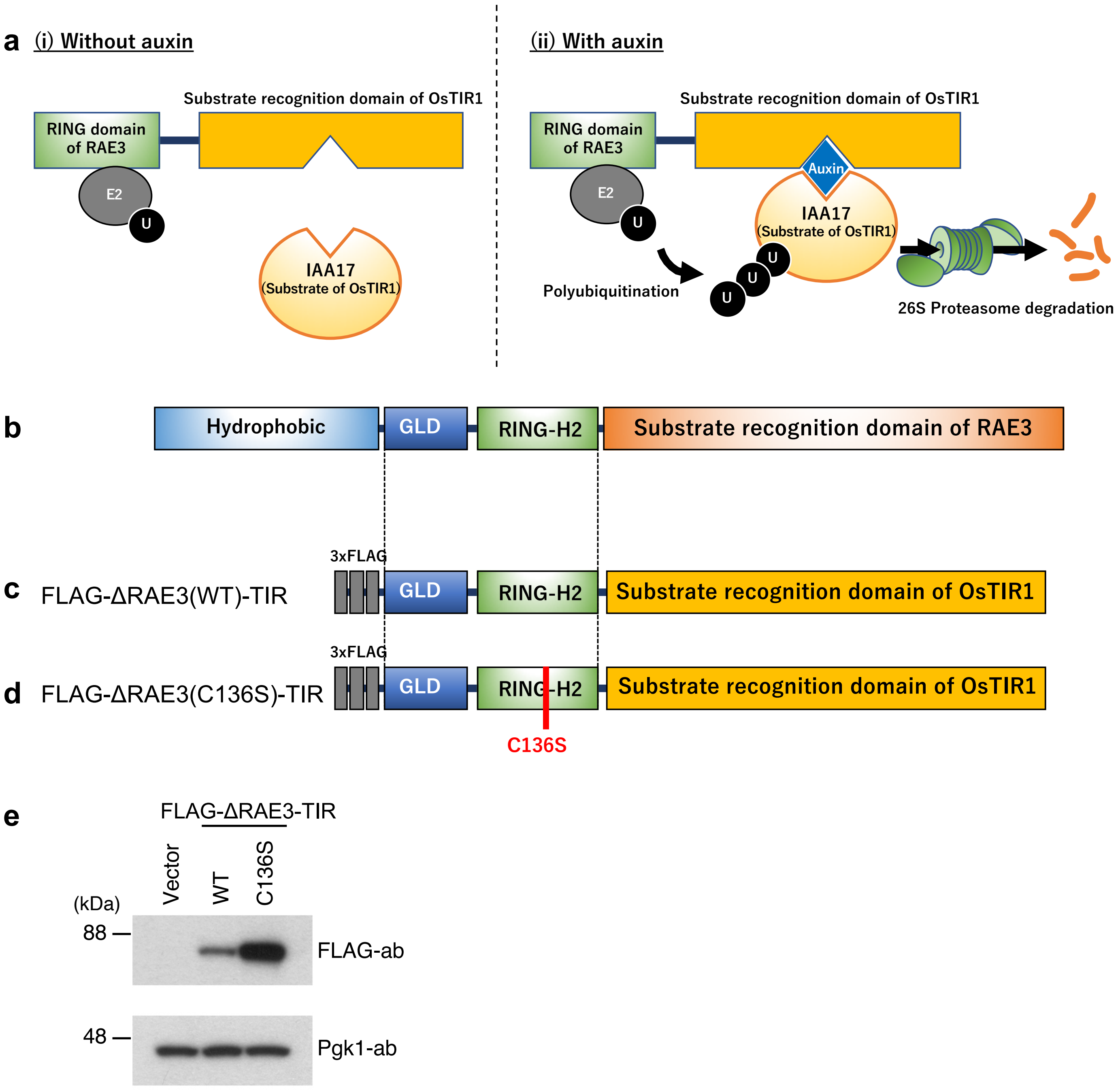

Figure S8. The establishment of auxin-inducible degron (AID) system. (a) Diagram of the E3 ubiquitin ligase assay in yeast using RAE3-TIR1 chimeric protein in the AID system. IAA17 is a known substrate of TIR1. In the presence of auxin, IAA17 combines with TIR1 and is ubiquitinated by E3 ubiquitin ligase for degradation through the 26S proteasome pathway. (b) Wild-type RAE3 structure, composed of a hydrophobic region at the N-terminus, GLD motif, RING-H2 domain and substrate recognition domain at the C-terminus. (c) Modified RAE3 construct designated as FLAG-ΔRAE3(WT)-TIR used for the E3 ubiquitin ligase assay in yeast. The hydrophobic region was truncated and fused with FLAG at the N-terminus. The OsTIR1 domain was swapped with the substrate recognition domain of RAE3. (d) Modified RAE3 construct with amino acid substitution in the RING-H2 domain was named FLAG-ΔRAE3(C136S)-TIR. (e) The expression level of the recombinant protein in yeast. Ab, antibody. Pgk1 was used as loading control.

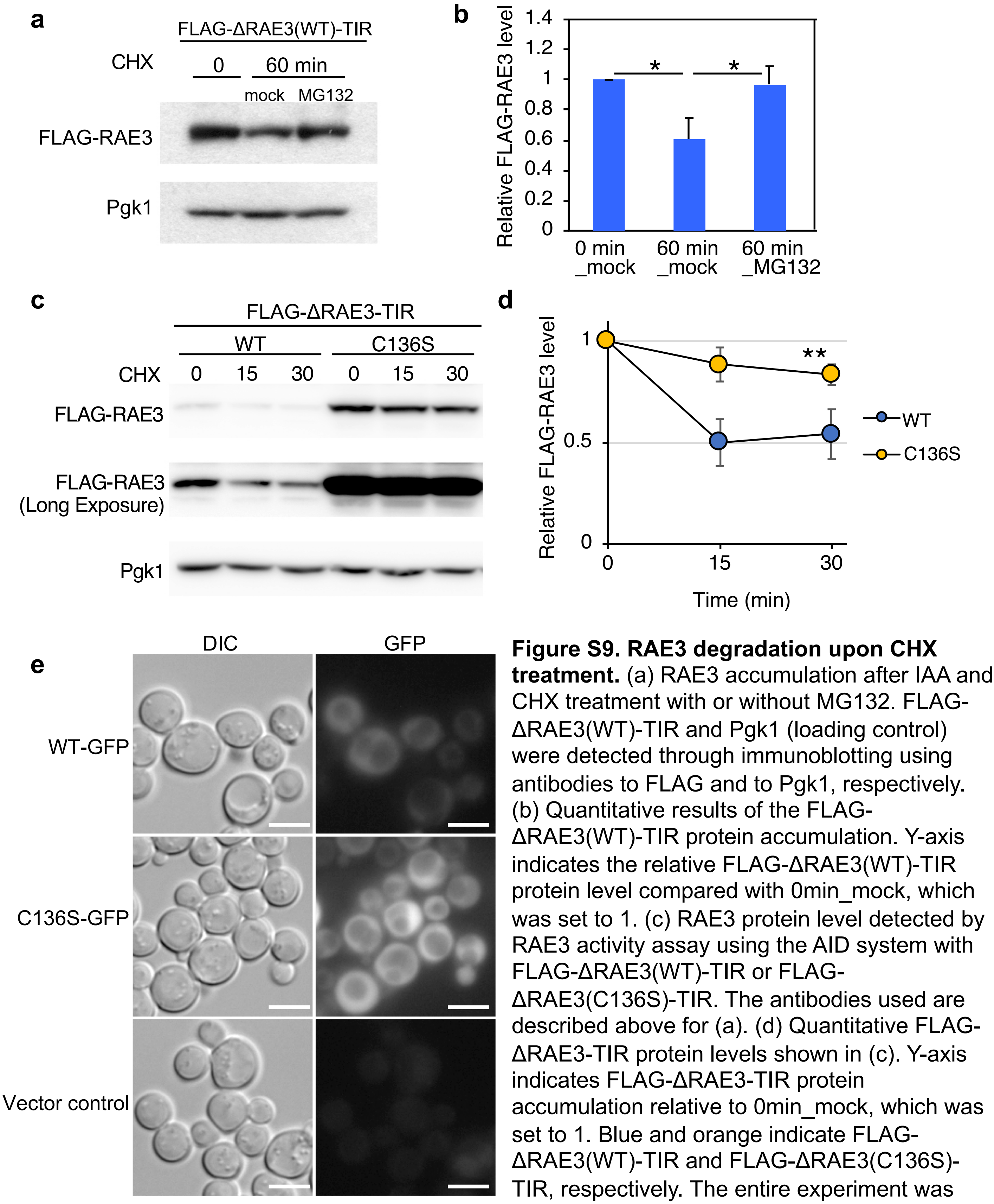

Figure S9. RAE3 degradation upon CHX treatment. (a) RAE3 accumulation after IAA and CHX treatment with or without MG132. FLAG-ΔRAE3(WT)-TIR and Pgk1 (loading control) were detected through immunoblotting using antibodies to FLAG and to Pgk1, respectively. (b) Quantitative results of the FLAG-ΔRAE3(WT)-TIR protein accumulation. Y-axis indicates the relative FLAG-ΔRAE3(WT)-TIR protein level compared with 0min_mock, which was set to 1. (c) RAE3 protein level detected by RAE3 activity assay using the AID system with FLAG-ΔRAE3(WT)-TIR or FLAG-ΔRAE3(C136S)-TIR. The antibodies used are described above for (a). (d) Quantitative FLAG-ΔRAE3-TIR protein levels shown in (c). Y-axis indicates FLAG-ΔRAE3-TIR protein accumulation relative to 0min_mock, which was set to 1. Blue and orange indicate FLAG-ΔRAE3(WT)-TIR and FLAG-ΔRAE3(C136S)-TIR, respectively. The entire experiment was replicated three times and scale bars represent standard deviations. Significance was tested by one-way analysis of variance (ANOVA) with Tukey's comparison. *P <0.05, ** P <0.01. (e) Expression pattern of ΔRAE3(WT)-TIR-GFP and ΔRAE3(C136S)-TIR1-GFP in yeast cells. Scale bars represent 5 μm.

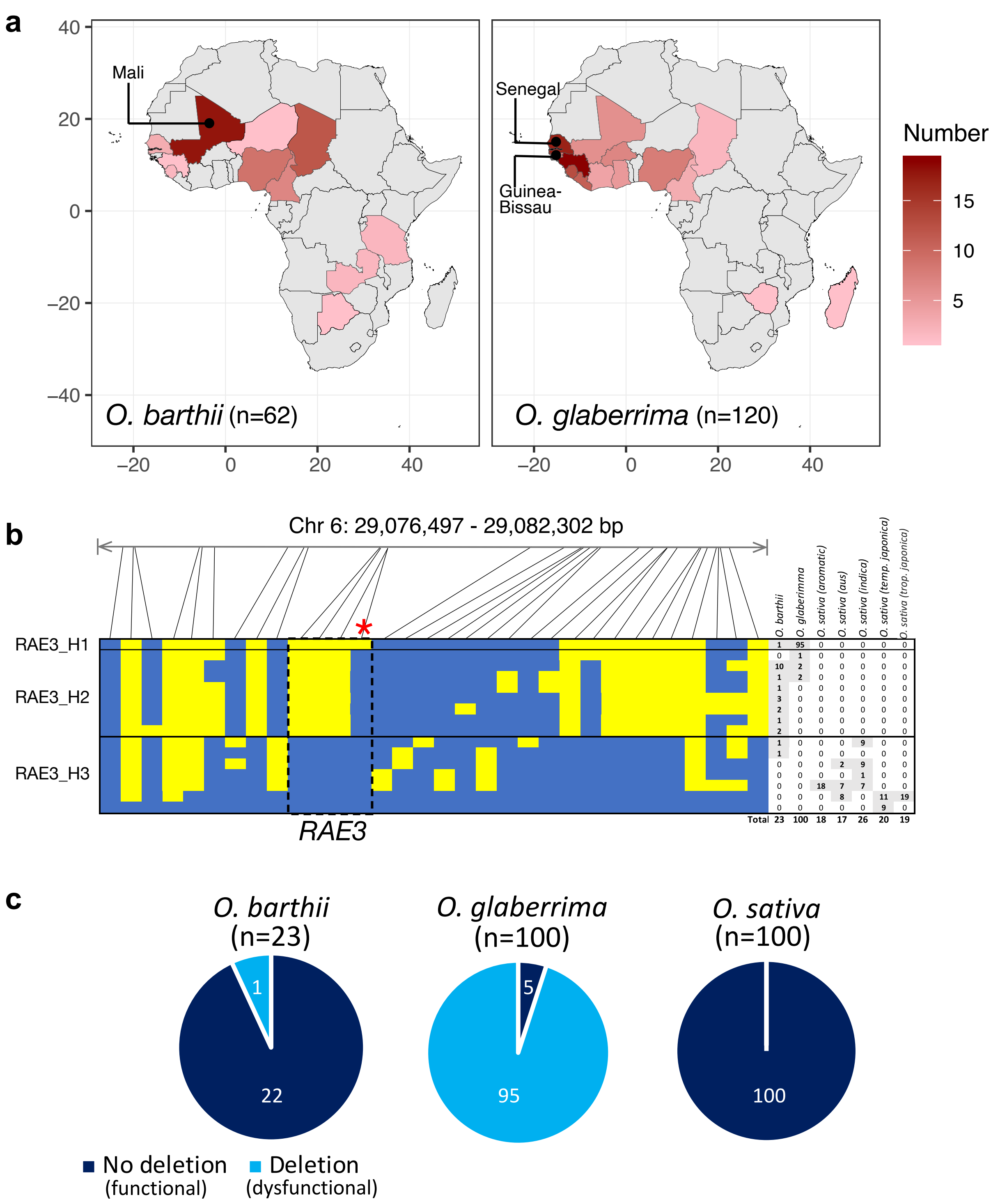

Figure S10. Geographical distribution of African rice accessions and haplotypes across *RAE3* region. (a) A total of 62 *O. barthii* and 120 *O. glaberrima* were called at the 48-bp indel from an assembled set of re-sequencing datasets aligned to cv. Nipponbare. Accessions originated from 22 countries primarily in West Africa listed in Table S2. Mali, Senegal and Guniea-Bissau are pointing because there are one *O. barthii* accession with deletion and five *O. glaberrima* accessions with non-deletion allele of *RAE3*. (b) Out of the African accessions that were called at the 48-bp indel and an additional set of *O. sativa*, haplotypes from 29,076,497-29,082,302bp on chromosome 6 were constructed. Accessions with greater than 10% missing calls were filtered out as were SNPs with greater than 0.05% missingness. Yellow and blue indicate alt (alteration) and reference respectively. The 223 individuals included in haplotype construction are found in Dataset S1. Red star indicates 48-bp deletion. (c) The accession ratio carrying 48-bp deletion in *O. barthii*, *O. glaberrima*, and *O. sativa*.

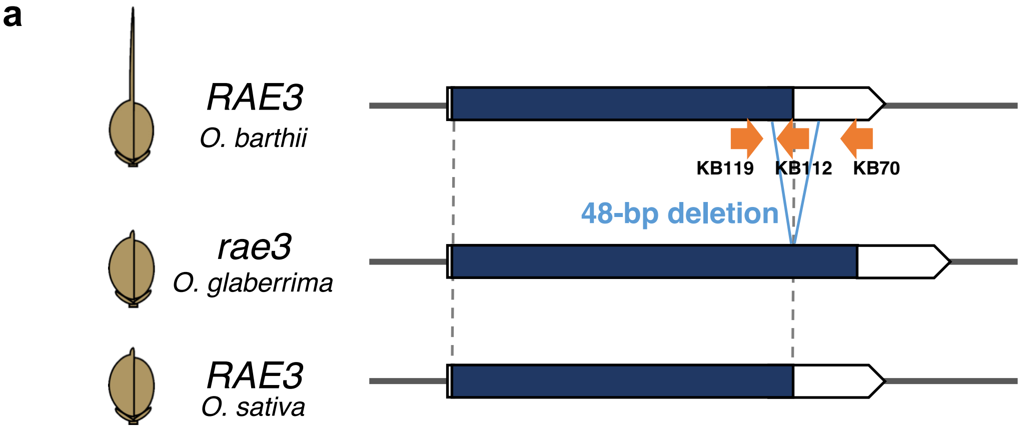

b

PCR amplicon size

Primer set \ Sample	*O. barthii*	*O. glaberrima*	*O. sativa*
KB119-KB70	963 bp	915 bp	960 bp
KB119-KB112	782 bp	No amplification	779 bp

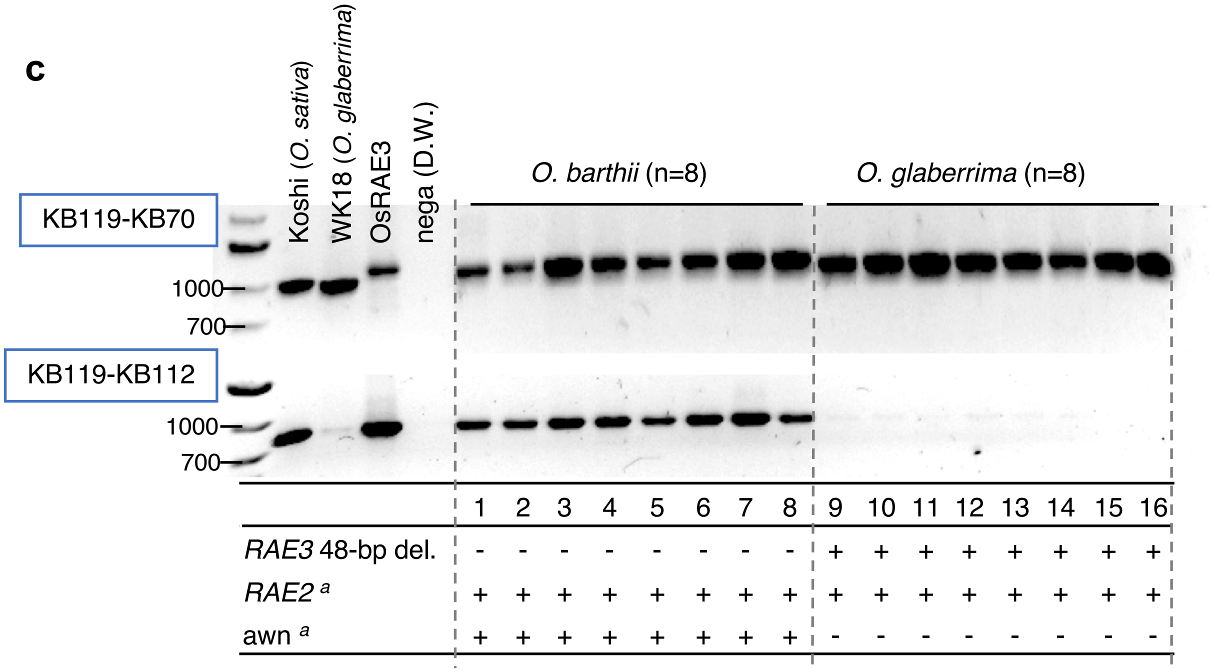

[a] RAE2 genotype and awn phenotype information were retrieved from previous report (16).

Figure S11. *RAE3* allele in the accessions used in the population analysis of *RAE2* research. (a) Primer location to detect 48-bp deletion in *RAE3* by PCR. (b) PCR amplicon size in each sample according to two primer sets. (c) Gel electrophoresis result and correspondence of the awn phenotype. Koshi, genomic DNA of *O. sativa japonica* cv. Koshihikari; WK18, genomic DNA of *O. glaberrima* acc. WK18; OsRAE3, plasmid carrying RAE3 CDS cloning from *O. sativa japonica* cv. Nipponbare; nega, negative control by distilled water (D.W.).

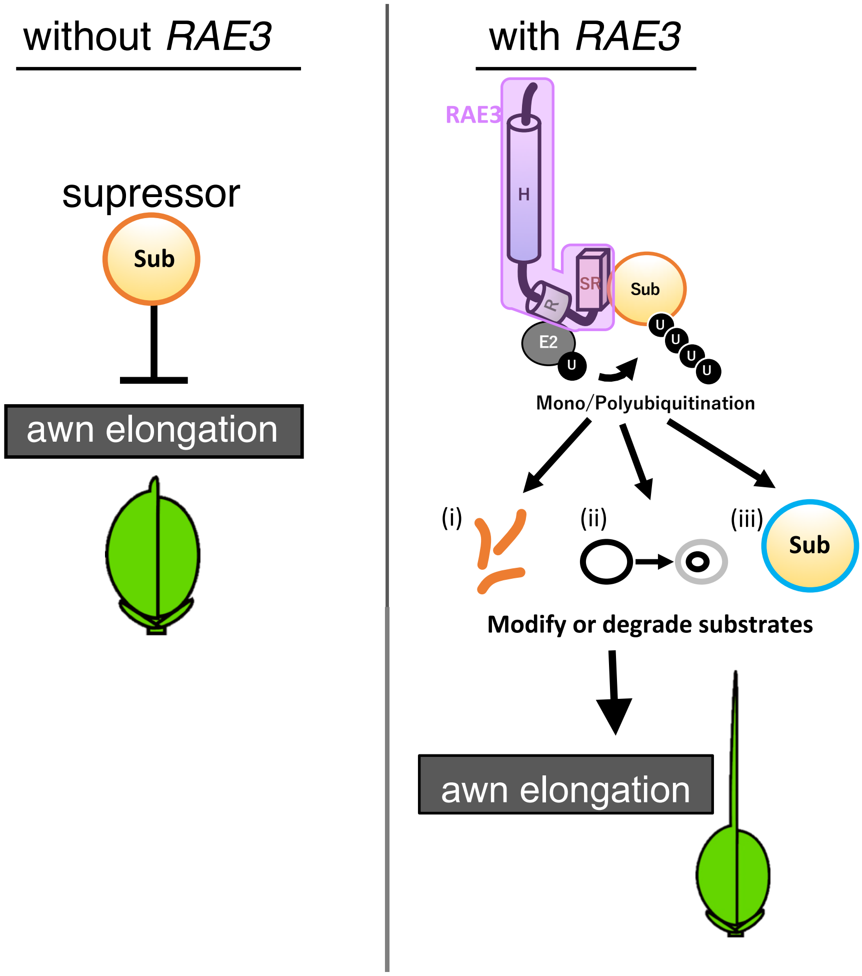

Figure S12. Model of *RAE3* function for awn elongation. Without functional RAE3, suppressor protein is present and negatively regulate awn elongation. In the presence of functional RAE3, the unknown suppressor protein would be ubiquitinated by RAE3 and (i) degraded through the 26S proteasome pathway, (ii) changed subcellular localization or (iii) modified protein surface features, then resulted in promoting the signal transduction of awn elongation.

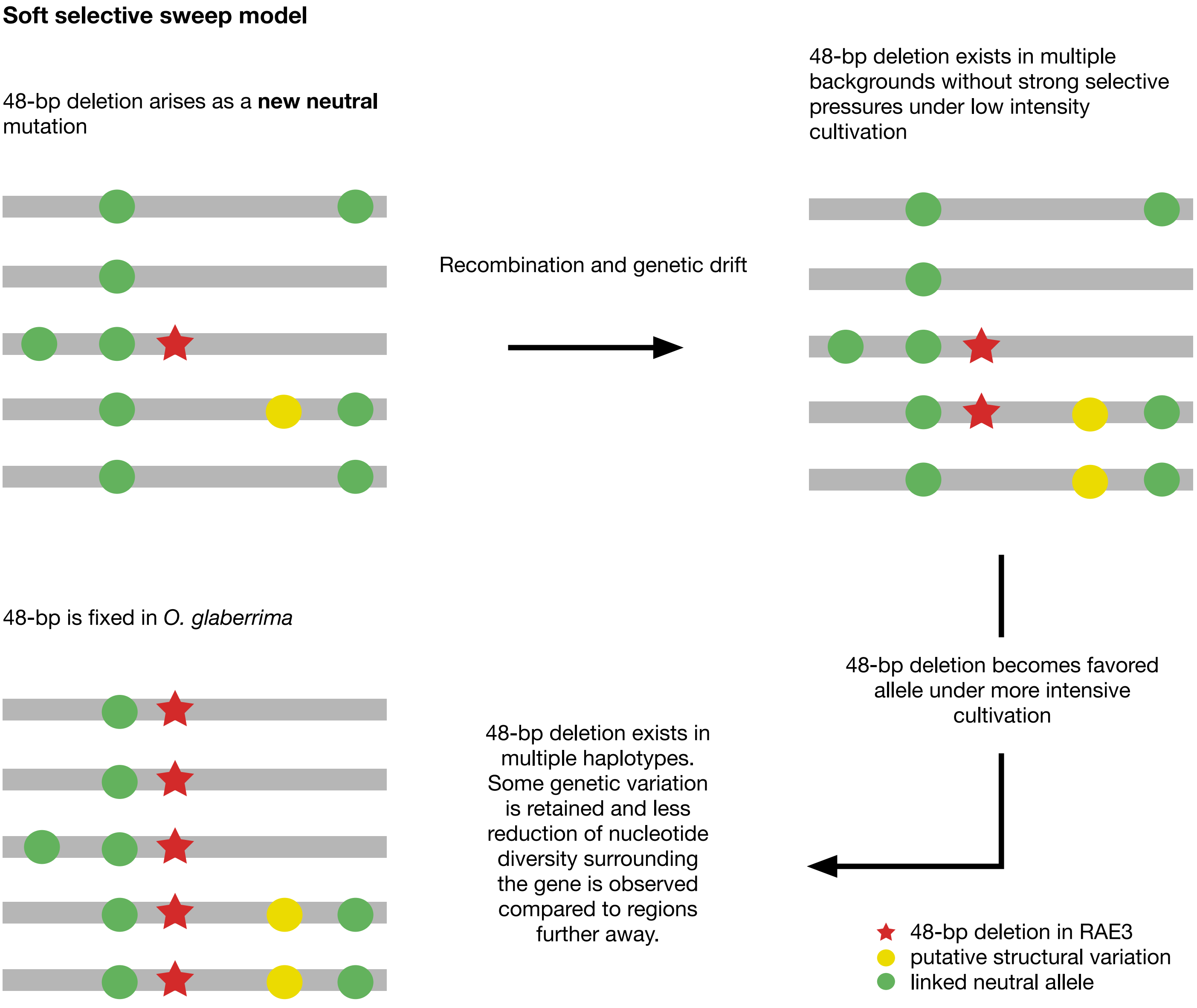

Figure S13. A model for soft selective sweep of the 48-bp deletion in *RAE3* in *O. glaberrima*. One model for how the functional 48-bp deletion in *RAE3* became fixed in *O. glaberrima* in two haplotypes (represented by putative structural variation found in re-sequencing data), whereby the deletion first arose as a neutral mutation in the early days of low intensity cultivation. In the model of a soft selective sweep, nucleotide diversity is reduced but not completed erased in the target region. Alternatively, a hard selective sweep would result in a very strong reduction in nucleotide diversity (π) surrounding *RAE3*, with increasing π values associated with increasing distance from the gene. Comparison of gene-based π with values across chromosome six showed that *RAE3* nucleotide diversity was at the 34th percentile of chromosome-wide values of π ratio between *O. glaberrima* and *O. barthii* (**Fig. 4b**).

Table S1. Awn phenotype data for each transgenic line.

	Number of seeds		Awned seed ratio per panicle (%)	awn length(mm)
	Awned	Awnless		
T65 (*O. sativa*)	0	137	0	0
OGBC4F1	NA	NA	NA	15.04 ± 2.97
WK18 (*O. glaberrima*)	0	101	0	0
2-03H (line1)	21	3	87.5	6.36 ± 1.69
2-03H (line2)	18	4	81.8	5.22 ± 1.38
2-03H (line3)	10	6	62.5	4.76 ± 1.07
V.C. (line1)	0	34	0	0
V.C. (line2)	0	37	0	0
V.C. (line3)	0	42	0	0
RAE3ox (line1)	59	0	100	8.71 ± 0.33
RAE3ox (line2)	62	4	93.9	7.34 ± 0.51
RAE3ox (line3)	58	0	100	10.5 ± 1.15
RAE3(WT)ox-4	19	5	79.2	3.25 ± 0.46
RAE3(WT)ox-6	24	3	88.9	3.37 ± 0.90
RAE3(WT)ox-15	20	3	87.0	3.91 ± 1.18
RAE3(C136S)ox-1	0	39	0	0
RAE3(C136S)ox-5	0	34	0	0
RAE3(C136S)ox-8	0	19	0	0

Table S2. Country of origin for African rice accessions shown in Figure S10a.

O. barthii

country	number
Botswana	1
Cameroon	7
Chad	12
Gambia	1
Guinea	1
Mali	18
Niger	1
Nigeria	9
Senegal	3
Sierra Leone	2
Tanzania	2
Unknown	3
Zambia	2
Total	62

O. glaberrima

country	number
Burkina Faso	7
Cameroon	3
Chad	2
Cote d'Ivoire	4
Gambia	1
Ghana	5
Guinea	19
Guinea-Bissau	8
Guyana	1
Liberia	11
Madagascar	1
Mali	6
NA	13
Nigeria	8
Senegal	17
Sierra Leone	13
Zimbabwe	1
Total	120

Table S3. Accessions used for diversity analyses.

accession	name	subpop	species	COUNTRY_OF_ORIGIN	set	group
B004	Qiuguang tentxi104hao	tej	*O. sativa*	Japan	3KGenome	temperate-japonica
B018	Meiguohuangkedao	trj	*O. sativa*	United States of America	3KGenome	tropical-japonica
B045	Gongchengxiang	tej	*O. sativa*	Japan	3KGenome	temperate-japonica
B046	Qiutianxiaoding	tej	*O. sativa*	Japan	3KGenome	temperate-japonica
B070	Laoguangtou83	tej	*O. sativa*	China	3KGenome	temperate-japonica
B160	Shuiyuan300li	tej	*O. sativa*	China	3KGenome	temperate-japonica
B162	Baigedao	tej	*O. sativa*	China	3KGenome	temperate-japonica
B182	Chimao	tej	*O. sativa*	Japan	3KGenome	temperate-japonica
B183	Qingnuo	tej	*O. sativa*	Japan	3KGenome	temperate-japonica
B190	ITA 221	trj	*O. sativa*	Nigeria	3KGenome	tropical-japonica
B204	Longhuamaohu	tej	*O. sativa*	China	3KGenome	temperate-japonica
B214	Xuanenchangtanqingzhan	ind2	*O. sativa*	China	3KGenome	indica
B250	AnnongwanjingB	tej	*O. sativa*	China	3KGenome	temperate-japonica
B269	Yuefu	tej	*O. sativa*	Japan	3KGenome	temperate-japonica
BODIAN_OUADEO	BODIAN_OUADEO	O. glaberrima	*O. glaberrima*	NA	AR_glab	O. glaberrima
CG_14	CG_14	O. glaberrima	*O. glaberrima*	NA	AR_glab	O. glaberrima
CX102	Tek Si Chut	ind1	*O. sativa*	Taiwan	3KGenome	indica
CX104	Sadri rice 1	aro	*O. sativa*	Iran	3KGenome	aromatic
CX106	SAL BUI BAO	trj	*O. sativa*	Viet Nam	3KGenome	tropical-japonica
CX111	Giza14	trj	*O. sativa*	Egypt	3KGenome	tropical-japonica
CX117	IR65600-27-1-2-2	ind1	*O. sativa*	Philippines	3KGenome	indica
CX143	Khasar	aro	*O. sativa*	Iran	3KGenome	aromatic
CX147	Seberang	ind2	*O. sativa*	Malaysia	3KGenome	indica
CX150	Chorofa	ind2	*O. sativa*	Philippines	3KGenome	indica
CX152	Cisadane	ind2	*O. sativa*	Indonesia	3KGenome	indica
CX162	TN1	ind1	*O. sativa*	Taiwan	3KGenome	indica
CX221	Zhongyouzao81	ind1	*O. sativa*	China	3KGenome	indica
CX269	IAC165	trj	*O. sativa*	Brazil	3KGenome	tropical-japonica
CX270	Taizhongbendi1hao	ind1	*O. sativa*	Taiwan	3KGenome	indica
CX417	Xa4+21	ind2	*O. sativa*	Philippines	3KGenome	indica
Gervex_2674	Gervex_2674	O. glaberrima	*O. glaberrima*	NA	AR_glab	O. glaberrima
IG_133	IG_133	O. glaberrima	*O. glaberrima*	NA	AR_glab	O. glaberrima
IG_48	IG_48	O. glaberrima	*O. glaberrima*	NA	AR_glab	O. glaberrima
IRIS_313-10097	MAEKJO	tej	*O. sativa*	Korea, Republic of	3KGenome	temperate-japonica
IRIS_313-10503	HA-GOO	ind1	*O. sativa*	China	3KGenome	indica
IRIS_313-10603	UCP122	aus	*O. sativa*	Bangladesh	3KGenome	aus
IRIS_313-10605	DV86	aus	*O. sativa*	Bangladesh	3KGenome	aus
IRIS_313-10642	SACHIKAZE	tej	*O. sativa*	Japan	3KGenome	temperate-japonica
IRIS_313-10670	ARC 10296	aro	*O. sativa*	India	3KGenome	aromatic
IRIS_313-10732	MADHUWA KARIA	aro	*O. sativa*	Nepal	3KGenome	aromatic
IRIS_313-10852	ARC 7336	aus	*O. sativa*	India	3KGenome	aus
IRIS_313-10861	ARC 11276	aus	*O. sativa*	India	3KGenome	aus
IRIS_313-10926	RAM SALEE KATAKA	aro	*O. sativa*	Nepal	3KGenome	aromatic
IRIS_313-10968	IH PEN SHIM MING	ind1	*O. sativa*	Brazil	3KGenome	indica
IRIS_313-11022	BASMATI SUFAID 100	aro	*O. sativa*	Pakistan	3KGenome	aromatic
IRIS_313-11026	HANSRAJ	aro	*O. sativa*	Pakistan	3KGenome	aromatic
IRIS_313-11037	421	aus	*O. sativa*	Pakistan	3KGenome	aus
IRIS_313-11039	KWANG-LU-AI 4	ind1	*O. sativa*	China	3KGenome	indica
IRIS_313-11045	ENGKABANG	trj	*O. sativa*	Malaysia	3KGenome	tropical-japonica
IRIS_313-11047	AUS 84	aus	*O. sativa*	Bangladesh	3KGenome	aus
IRIS_313-11048	AUS 171	aus	*O. sativa*	Bangladesh	3KGenome	aus
IRIS_313-11050	AUS 233	aus	*O. sativa*	Bangladesh	3KGenome	aus
IRIS_313-11051	AUS 242	aus	*O. sativa*	Bangladesh	3KGenome	aus
IRIS_313-11053	AUS 282	aus	*O. sativa*	Bangladesh	3KGenome	aus
IRIS_313-11056	AUS 301	aus	*O. sativa*	Bangladesh	3KGenome	aus
IRIS_313-11057	AUS 308	aus	*O. sativa*	Bangladesh	3KGenome	aus
IRIS_313-11058	AUS 329	aus	*O. sativa*	Bangladesh	3KGenome	aus
IRIS_313-11064	BORO 275	aus	*O. sativa*	Bangladesh	3KGenome	aus
IRIS_313-11102		trj	*O. sativa*	Liberia	3KGenome	tropical-japonica
IRIS_313-11103		trj	*O. sativa*	Liberia	3KGenome	tropical-japonica
IRIS_313-11104		trj	*O. sativa*	Liberia	3KGenome	tropical-japonica
IRIS_313-11202	4583	tej	*O. sativa*	China	3KGenome	temperate-japonica
IRIS_313-11238	IAC1132	trj	*O. sativa*	Brazil	3KGenome	tropical-japonica
IRIS_313-11265	ARC 14150	aus	*O. sativa*	India	3KGenome	aus
IRIS_313-11277	ARC 15129	aus	*O. sativa*	India	3KGenome	aus
IRIS_313-11298	ARC 13544	aus	*O. sativa*	India	3KGenome	aus
IRIS_313-11350	BAJAL	aro	*O. sativa*	India	3KGenome	aromatic
IRIS_313-11356	CR44-1	aro	*O. sativa*	India	3KGenome	aromatic
IRIS_313-11362	KEYA NUNIA	aro	*O. sativa*	India	3KGenome	aromatic
IRIS_313-11373	T 3	aro	*O. sativa*	India	3KGenome	aromatic
IRIS_313-11401	MOTANGA	aro	*O. sativa*	Bangladesh	3KGenome	aromatic
IRIS_313-11436	SAKAZELE(538)	trj	*O. sativa*	Cote d'Ivoire	3KGenome	tropical-japonica

Table S3. Accessions used for diversity analyses. (continued)

accession	name	subpop	species	COUNTRY_OF_ORIGIN	set	group
IRIS_313-11626	GUDURA	aro	*O. sativa*	Nepal	3KGenome	aromatic
IRIS_313-11630	POHHERLIMASION	aro	*O. sativa*	Nepal	3KGenome	aromatic
IRIS_313-11645	NCS766	ind3	*O. sativa*	India	3KGenome	indica
IRIS_313-11652	GONG SHE 9	tej	*O. sativa*	China	3KGenome	temperate-japonica
IRIS_313-11657	EX WUKARI(WILD)	ind3	*O. sativa*	Nigeria	3KGenome	indica
IRIS_313-11659	TOBOHUN	trj	*O. sativa*	Sierra Leone	3KGenome	tropical-japonica
IRIS_313-11661	91-382	tej	*O. sativa*	Bhutan	3KGenome	temperate-japonica
IRIS_313-11665	JIN HUA 258	ind1	*O. sativa*	China	3KGenome	indica
IRIS_313-11667	LUO AI ZAO 3	ind1	*O. sativa*	China	3KGenome	indica
IRIS_313-11671	DERAWA	ind3	*O. sativa*	Nepal	3KGenome	indica
IRIS_313-11673	BURI-BURING	trj	*O. sativa*	Philippines	3KGenome	tropical-japonica
IRIS_313-11689	DECHANGBYEO	tej	*O. sativa*	Korea, Republic of	3KGenome	temperate-japonica
IRIS_313-11717	JATI MANI	ind2	*O. sativa*	Indonesia	3KGenome	indica
IRIS_313-11723	DISSIGBE	ind3	*O. sativa*	Guinea	3KGenome	indica
IRIS_313-11725	ZUIHOU	tej	*O. sativa*	Japan	3KGenome	temperate-japonica
IRIS_313-11736	MALAGKIT(PINELIPE)	trj	*O. sativa*	Philippines	3KGenome	tropical-japonica
IRIS_313-11739	NAKPUI	trj	*O. sativa*	Ghana	3KGenome	tropical-japonica
IRIS_313-11755	A 2-257	trj	*O. sativa*	Liberia	3KGenome	tropical-japonica
IRIS_313-11756	BENGALI VAKAHANA 1448	trj	*O. sativa*	Madagascar	3KGenome	tropical-japonica
IRIS_313-11759	K 2 C 45	trj	*O. sativa*	Cote d'Ivoire	3KGenome	tropical-japonica
IRIS_313-11789	SABORAMANDALOAFO	ind3	*O. sativa*	Madagascar	3KGenome	indica
IRIS_313-11800	FAN GENG 6	tej	*O. sativa*	China	3KGenome	temperate-japonica
IRIS_313-11809	MHARAKA	aus	*O. sativa*	Kenya	3KGenome	aus
IRIS_313-11821	19	ind3	*O. sativa*	India	3KGenome	indica
IRIS_313-11823	BHU BHUSI	ind3	*O. sativa*	India	3KGenome	indica
IRIS_313-11824	DHANIYA PHOOL	ind3	*O. sativa*	India	3KGenome	indica
IRIS_313-11825	HANSRAJ	aro	*O. sativa*	India	3KGenome	aromatic
IRIS_313-11929	GILINGAN MARANGRAS	trj	*O. sativa*	Philippines	3KGenome	tropical-japonica
IRIS_313-12094	KASHA	aro	*O. sativa*	Bangladesh	3KGenome	aromatic
IRIS_313-15908	COLOMBIA XXI	aus	*O. sativa*	Colombia	3KGenome	aus
IRIS_313-7808	WAS 173-B-B-6-2-2	ind2	*O. sativa*	Senegal	3KGenome	indica
IRIS_313-7914	IRAT 112	trj	*O. sativa*	Cote d'Ivoire	3KGenome	tropical-japonica
IRIS_313-8312	BATU	ind2	*O. sativa*	Malaysia	3KGenome	indica
IRIS_313-8326	JC1	aro	*O. sativa*	India	3KGenome	aromatic
IRIS_313-8391	MARAGBE	ind3	*O. sativa*	Burkina Faso	3KGenome	indica
IRIS_313-8656	BASMATI 1	aro	*O. sativa*	Pakistan	3KGenome	aromatic
IRIS_313-8747	DOM-ZARD	aro	*O. sativa*	Iran	3KGenome	aromatic
IRIS_313-8755	NORIN 6	tej	*O. sativa*	Japan	3KGenome	temperate-japonica
IRIS_313-8813	ARC 10497	aro	*O. sativa*	India	3KGenome	aromatic
IRIS_313-8864	NOROI	aus	*O. sativa*	Bangladesh	3KGenome	aus
IRIS_313-8930	MUKKALA BAZAL	ind2	*O. sativa*	Bangladesh	3KGenome	indica
IRIS_313-9020	LEUANG CHAIYAPHUM	ind2	*O. sativa*	Thailand	3KGenome	indica
IRIS_313-9294	AUS 78-62	ind3	*O. sativa*	Gambia	3KGenome	indica
SAMN02142780	IRGC105895	nivara	*O. nivara*	Bangladesh	125Genome	ORSC
SAMN02142781	YJ	rufipogon	*O. rufipogon*	China	125Genome	ORSC
SAMN02142782	IRGC106413	rufipogon	*O. rufipogon*	Vietnam	125Genome	ORSC
SAMN02142783	IRGC103818	rufipogon	*O. rufipogon*	China	125Genome	ORSC
SAMN02142784	IRGC104624	rufipogon	*O. rufipogon*	China	125Genome	ORSC
SAMN02142785	IRGC103813	nivara	*O. nivara*	China	125Genome	ORSC
SAMN02142786	Khao_Pa	nivara	*O. nivara*	Laos	125Genome	ORSC
SAMN02142787	P46	rufipogon	*O. rufipogon*	China	125Genome	ORSC
SAMN02142788	YW1944	rufipogon	*O. rufipogon*	China	125Genome	ORSC
SAMN02142789	CA_97-053	rufipogon	*O. rufipogon*	Cambodia	125Genome	ORSC
SAMN02142790	HK_47	rufipogon	*O. rufipogon*	India	125Genome	ORSC
SAMN02142791	Sahul	rufipogon	*O. rufipogon*	India	125Genome	ORSC
SAMN02142792	HAISHA_CAMAN	rufipogon	*O. rufipogon*	Bangladesh	125Genome	ORSC
SAMN02142793	L89-12	rufipogon	*O. rufipogon*	Laos	125Genome	ORSC
SAMN02142794	IRGC105843	rufipogon	*O. rufipogon*	Thailand	125Genome	ORSC
SAMN02142795	MV_89-80	rufipogon	*O. rufipogon*	India	125Genome	ORSC
SAMN02142796	PADI_PADIAN	rufipogon	*O. rufipogon*	Indonesia	125Genome	ORSC
SAMN02142797	042.87.34	rufipogon	*O. rufipogon*	India	125Genome	ORSC
SAMN02142798	DAL_DHAN	rufipogon	*O. rufipogon*	Bangladesh	125Genome	ORSC
SAMN02142799	YOC4	rufipogon	*O. rufipogon*	Nepal	125Genome	ORSC
SAMN02142800	IRGC105567	rufipogon	*O. rufipogon*	Indonesia	125Genome	ORSC
SAMN02142801	IRGC105494	rufipogon	*O. rufipogon*	Myanmar	125Genome	ORSC
SAMN02142802	IRGC103841	nivara	*O. nivara*	Bangladesh	125Genome	ORSC
SAMN02142803	IRGC100918	nivara	*O. nivara*	Cambodia	125Genome	ORSC
SAMN02142804	W1943	rufipogon	*O. rufipogon*	China	125Genome	ORSC
SAMN02142805	W1945	rufipogon	*O. rufipogon*	China	125Genome	ORSC
SAMN02142806	IRGC100900	nivara	*O. nivara*	India	125Genome	ORSC
SAMN02142807	W1944	rufipogon	*O. rufipogon*	China	125Genome	ORSC
SAMN02142808	IRGC103821	nivara	*O. nivara*	China	125Genome	ORSC

Table S3. Accessions used for diversity analyses. (continued)

accession	name	subpop	species	COUNTRY_OF_ORIGIN	set	group
SAMN02142809	IRGC105349	rufipogon	*O. rufipogon*	India	125Genome	ORSC
SAMN02142810	IRGC101193	nivara	*O. nivara*	Taiwan	125Genome	ORSC
SAMN02142811	IRGC105431	nivara	*O. nivara*	Sri Lanka	125Genome	ORSC
SAMN02142812	IRGC105491	rufipogon	*O. rufipogon*	Malaysia	125Genome	ORSC
SAMN02142813	IRGC81881	rufipogon	*O. rufipogon*	India	125Genome	ORSC
SAMN02142814	IRGC100203	rufipogon	*O. rufipogon*	Myanmar	125Genome	ORSC
SAMN02142815	IRGC80433	rufipogon	*O. rufipogon*	India	125Genome	ORSC
SAMN02142816	IRGC93224	rufipogon	*O. rufipogon*	Nepal	125Genome	ORSC
SAMN02142854	OR44	meridionalis	*O. meridionalis*	Australia	125Genome	ORSC
SAMN02142855	punctata	punctata	*O. punctata*	NA	125Genome	ORSC
SAMN02142856	IRGC105220	officinalis	*O. officinalis*	Indonesia	125Genome	ORSC
SRR063624	Yuan 3-9	rufipogon	*wild rice*	Yunnan, China	Xu	ORSC
SRR1206500	IRGC103469	NA	*O. glaberrima*	Burkina Faso	Wing	O. glaberrima
SRR1206501	TOG5457	NA	*O. glaberrima*	Nigeria	Wing	O. glaberrima
SRR1206502	TOG5467	NA	*O. glaberrima*	Nigeria	Wing	O. glaberrima
SRR1206503	TOG5923	NA	*O. glaberrima*	Liberia	Wing	O. glaberrima
SRR1206504	TOG5949	NA	*O. glaberrima*	Liberia	Wing	O. glaberrima
SRR1206505	TOG7025	NA	*O. glaberrima*	Sierra Leone	Wing	O. glaberrima
SRR1206506	TOG7102	NA	*O. glaberrima*	Mali	Wing	O. glaberrima
SRR1206507	IRGC101049	NA	*O. glaberrima*	Senegal	Wing	O. glaberrima
SRR1206508	IRGC103472	NA	*O. glaberrima*	Burkina Faso	Wing	O. glaberrima
SRR1206509	IRGC103520	NA	*O. glaberrima*	Mali	Wing	O. glaberrima
SRR1206510	IRGC103632	NA	*O. glaberrima*	Mali	Wing	O. glaberrima
SRR1206511	IRGC103937	NA	*O. glaberrima*	Liberia	Wing	O. glaberrima
SRR1206512	IRGC104206	NA	*O. glaberrima*	Ghana	Wing	O. glaberrima
SRR1206513	IRGC104574	NA	*O. glaberrima*	Mali	Wing	O. glaberrima
SRR1206514	IRGC104955	NA	*O. glaberrima*	Sierra Leone	Wing	O. glaberrima
SRR1206515	IRGC67563	NA	*O. glaberrima*	Ghana	Wing	O. glaberrima
SRR1206516	IRGC68939	NA	*O. glaberrima*	Madagascar	Wing	O. glaberrima
SRR1206517	IRGC68976	NA	*O. glaberrima*	Guyana	Wing	O. glaberrima
SRR1206518	IRGC75500	NA	*O. glaberrima*	Burkina Faso	Wing	O. glaberrima
SRR1206519	IRGC96841	NA	*O. glaberrima*	Zimbabwe	Wing	O. glaberrima
SRR3231659	IRGC 103442	NA	*O. glaberrima*	Senegal	Purugganan	O. glaberrima
SRR3231660	IRGC 103450	NA	*O. glaberrima*	Gambia	Purugganan	O. glaberrima
SRR3231661	IRGC 103452	NA	*O. glaberrima*	Senegal	Purugganan	O. glaberrima
SRR3231662	IRGC 103456	NA	*O. glaberrima*	Senegal	Purugganan	O. glaberrima
SRR3231663	IRGC 103461	NA	*O. glaberrima*	Senegal	Purugganan	O. glaberrima
SRR3231664	IRGC 103463	NA	*O. glaberrima*	Senegal	Purugganan	O. glaberrima
SRR3231665	IRGC 103517	NA	*O. glaberrima*	Mali	Purugganan	O. glaberrima
SRR3231666	IRGC 103530	NA	*O. glaberrima*	Mali	Purugganan	O. glaberrima
SRR3231667	IRGC 103592	NA	*O. glaberrima*	Cameroon	Purugganan	O. glaberrima
SRR3231668	IRGC 103599	NA	*O. glaberrima*	Cameroon	Purugganan	O. glaberrima
SRR3231669	IRGC 103922	NA	*O. glaberrima*	Nigeria	Purugganan	O. glaberrima
SRR3231670	IRGC 103937	NA	*O. glaberrima*	Liberia	Purugganan	O. glaberrima
SRR3231671	IRGC 103946	NA	*O. glaberrima*	Liberia	Purugganan	O. glaberrima
SRR3231672	IRGC 103948	NA	*O. glaberrima*	Liberia	Purugganan	O. glaberrima
SRR3231673	IRGC 103949	NA	*O. glaberrima*	Liberia	Purugganan	O. glaberrima
SRR3231674	IRGC 103953	NA	*O. glaberrima*	Sierra Leone	Purugganan	O. glaberrima
SRR3231675	IRGC 103955	NA	*O. glaberrima*	Senegal	Purugganan	O. glaberrima
SRR3231676	IRGC 103956	NA	*O. glaberrima*	Senegal	Purugganan	O. glaberrima
SRR3231677	IRGC 103957	NA	*O. glaberrima*	Senegal	Purugganan	O. glaberrima
SRR3231678	IRGC 103958	NA	*O. glaberrima*	Senegal	Purugganan	O. glaberrima
SRR3231679	IRGC 103959	NA	*O. glaberrima*	Senegal	Purugganan	O. glaberrima
SRR3231680	IRGC 103960	NA	*O. glaberrima*	Senegal	Purugganan	O. glaberrima
SRR3231681	IRGC 103963	NA	*O. glaberrima*	Senegal	Purugganan	O. glaberrima
SRR3231682	IRGC 103967	NA	*O. glaberrima*	Senegal	Purugganan	O. glaberrima
SRR3231683	IRGC 103981	NA	*O. glaberrima*	Nigeria	Purugganan	O. glaberrima
SRR3231684	IRGC 103982	NA	*O. glaberrima*	Nigeria	Purugganan	O. glaberrima
SRR3231685	IRGC 103988	NA	*O. glaberrima*	Sierra Leone	Purugganan	O. glaberrima
SRR3231686	IRGC 103989	NA	*O. glaberrima*	Sierra Leone	Purugganan	O. glaberrima
SRR3231687	IRGC 103991	NA	*O. glaberrima*	Sierra Leone	Purugganan	O. glaberrima
SRR3231688	IRGC 103992	NA	*O. glaberrima*	Sierra Leone	Purugganan	O. glaberrima
SRR3231689	IRGC 103993	NA	*O. glaberrima*	Sierra Leone	Purugganan	O. glaberrima
SRR3231690	IRGC 103994	NA	*O. glaberrima*	Sierra Leone	Purugganan	O. glaberrima
SRR3231691	IRGC 103995	NA	*O. glaberrima*	Sierra Leone	Purugganan	O. glaberrima
SRR3231692	IRGC 104011	NA	*O. glaberrima*	Nigeria	Purugganan	O. glaberrima
SRR3231693	IRGC 104022	NA	*O. glaberrima*	Guinea-Bissau	Purugganan	O. glaberrima
SRR3231694	IRGC 104023	NA	*O. glaberrima*	Guinea-Bissau	Purugganan	O. glaberrima
SRR3231695	IRGC 104024	NA	*O. glaberrima*	Guinea-Bissau	Purugganan	O. glaberrima
SRR3231696	IRGC 104025	NA	*O. glaberrima*	Guinea-Bissau	Purugganan	O. glaberrima
SRR3231697	IRGC 104028	NA	*O. glaberrima*	Guinea-Bissau	Purugganan	O. glaberrima
SRR3231698	IRGC 104029	NA	*O. glaberrima*	Guinea-Bissau	Purugganan	O. glaberrima

Table S3. Accessions used for diversity analyses. (continued)

accession	name	subpop	species	COUNTRY_OF_ORIGIN	set	group
SRR3231699	IRGC 104030	NA	*O. glaberrima*	Guinea-Bissau	Purugganan	O. glaberrima
SRR3231700	IRGC 104032	NA	*O. glaberrima*	Guinea-Bissau	Purugganan	O. glaberrima
SRR3231701	IRGC 104034	NA	*O. glaberrima*	Cote d'Ivoire	Purugganan	O. glaberrima
SRR3231702	IRGC 104035	NA	*O. glaberrima*	Cote d'Ivoire	Purugganan	O. glaberrima
SRR3231703	IRGC 104036	NA	*O. glaberrima*	Cote d'Ivoire	Purugganan	O. glaberrima
SRR3231704	IRGC 104044	NA	*O. glaberrima*	Chad	Purugganan	O. glaberrima
SRR3231705	IRGC 104047	NA	*O. glaberrima*	Cameroon	Purugganan	O. glaberrima
SRR3231706	IRGC 104165	NA	*O. glaberrima*	Guinea	Purugganan	O. glaberrima
SRR3231707	IRGC 104173	NA	*O. glaberrima*	Guinea	Purugganan	O. glaberrima
SRR3231708	IRGC 104177	NA	*O. glaberrima*	Guinea	Purugganan	O. glaberrima
SRR3231709	IRGC 104178	NA	*O. glaberrima*	Guinea	Purugganan	O. glaberrima
SRR3231710	IRGC 104180	NA	*O. glaberrima*	Guinea	Purugganan	O. glaberrima
SRR3231711	IRGC 104181	NA	*O. glaberrima*	Guinea	Purugganan	O. glaberrima
SRR3231712	IRGC 104182	NA	*O. glaberrima*	Guinea	Purugganan	O. glaberrima
SRR3231713	IRGC 104187	NA	*O. glaberrima*	Guinea	Purugganan	O. glaberrima
SRR3231714	IRGC 104190	NA	*O. glaberrima*	Guinea	Purugganan	O. glaberrima
SRR3231715	IRGC 104194	NA	*O. glaberrima*	Guinea	Purugganan	O. glaberrima
SRR3231716	IRGC 104195	NA	*O. glaberrima*	Ghana	Purugganan	O. glaberrima
SRR3231717	IRGC 104231	NA	*O. glaberrima*	Sierra Leone	Purugganan	O. glaberrima
SRR3231718	IRGC 104260	NA	*O. glaberrima*	Ghana	Purugganan	O. glaberrima
SRR3231719	IRGC 104294	NA	*O. glaberrima*	Chad	Purugganan	O. glaberrima
SRR3231720	IRGC 104533	NA	*O. glaberrima*	Nigeria	Purugganan	O. glaberrima
SRR3231721	IRGC 104545	NA	*O. glaberrima*	Nigeria	Purugganan	O. glaberrima
SRR3231722	IRGC 104561	NA	*O. glaberrima*	Sierra Leone	Purugganan	O. glaberrima
SRR3231723	IRGC 104562	NA	*O. glaberrima*	Sierra Leone	Purugganan	O. glaberrima
SRR3231724	IRGC 104566	NA	*O. glaberrima*	Senegal	Purugganan	O. glaberrima
SRR3231725	IRGC 104571	NA	*O. glaberrima*	Senegal	Purugganan	O. glaberrima
SRR3231726	IRGC 104573	NA	*O. glaberrima*	Cote d'Ivoire	Purugganan	O. glaberrima
SRR3231727	IRGC 104595	NA	*O. glaberrima*	Mali	Purugganan	O. glaberrima
SRR3231728	IRGC 104904	NA	*O. glaberrima*	Nigeria	Purugganan	O. glaberrima
SRR3231729	IRGC 104934	NA	*O. glaberrima*	Burkina Faso	Purugganan	O. glaberrima
SRR3231730	IRGC 105005	NA	*O. glaberrima*	Guinea	Purugganan	O. glaberrima
SRR3231731	IRGC 105011	NA	*O. glaberrima*	Guinea	Purugganan	O. glaberrima
SRR3231732	IRGC 105021	NA	*O. glaberrima*	Guinea	Purugganan	O. glaberrima
SRR3231733	IRGC 105026	NA	*O. glaberrima*	Guinea	Purugganan	O. glaberrima
SRR3231734	IRGC 105034	NA	*O. glaberrima*	Guinea	Purugganan	O. glaberrima
SRR3231735	IRGC 105036	NA	*O. glaberrima*	Guinea	Purugganan	O. glaberrima
SRR3231736	IRGC 105038	NA	*O. glaberrima*	Guinea	Purugganan	O. glaberrima
SRR3231737	IRGC 105043	NA	*O. glaberrima*	Guinea	Purugganan	O. glaberrima
SRR3231738	IRGC 105044	NA	*O. glaberrima*	Guinea	Purugganan	O. glaberrima
SRR3231739	IRGC 105048	NA	*O. glaberrima*	Liberia	Purugganan	O. glaberrima
SRR3231740	IRGC 105049	NA	*O. glaberrima*	Liberia	Purugganan	O. glaberrima
SRR3231741	IRGC 105050	NA	*O. glaberrima*	Liberia	Purugganan	O. glaberrima
SRR3231742	IRGC 105052	NA	*O. glaberrima*	Guinea	Purugganan	O. glaberrima
SRR3231743	IRGC 58622	NA	*O. glaberrima*	Sierra Leone	Purugganan	O. glaberrima
SRR3231744	IRGC 61457	NA	*O. glaberrima*	Liberia	Purugganan	O. glaberrima
SRR3231745	IRGC 67563	NA	*O. glaberrima*	Ghana	Purugganan	O. glaberrima
SRR3231746	IRGC 75546	NA	*O. glaberrima*	Burkina Faso	Purugganan	O. glaberrima
SRR3231747	IRGC 75618	NA	*O. glaberrima*	Burkina Faso	Purugganan	O. glaberrima
SRR3231748	IRGC 75729	NA	*O. glaberrima*	Burkina Faso	Purugganan	O. glaberrima
SRR3231749	TOg6203	NA	*O. glaberrima*	Guinea	Purugganan	O. glaberrima
SRR3231750	TOg7135	NA	*O. glaberrima*	Senegal	Purugganan	O. glaberrima
SRR3231751	TOg7197	NA	*O. glaberrima*	Cote d'Ivoire	Purugganan	O. glaberrima
TOG_14367	TOG_14367	O. glaberrima	*O. glaberrima*	NA	AR_glab	O. glaberrima
TOG_16704	TOG_16704	O. glaberrima	*O. glaberrima*	NA	AR_glab	O. glaberrima
TOG_5485	TOG_5485	O. glaberrima	*O. glaberrima*	NA	AR_glab	O. glaberrima
TOG_5505_A	TOG_5505_A	O. glaberrima	*O. glaberrima*	NA	AR_glab	O. glaberrima
TOG_5681	TOG_5681	O. glaberrima	*O. glaberrima*	NA	AR_glab	O. glaberrima
TOG_5980_A	TOG_5980_A	O. glaberrima	*O. glaberrima*	NA	AR_glab	O. glaberrima
TOG_6218_B	TOG_6218_B	O. glaberrima	*O. glaberrima*	NA	AR_glab	O. glaberrima
TOG_6519_A	TOG_6519_A	O. glaberrima	*O. glaberrima*	NA	AR_glab	O. glaberrima
TOG_6520	TOG_6520	O. glaberrima	*O. glaberrima*	NA	AR_glab	O. glaberrima
TOG_7148	TOG_7148	O. glaberrima	*O. glaberrima*	NA	AR_glab	O. glaberrima
TOG_7206	TOG_7206	O. glaberrima	*O. glaberrima*	NA	AR_glab	O. glaberrima
TOG_7250_A	TOG_7250_A	O. glaberrima	*O. glaberrima*	NA	AR_glab	O. glaberrima
TOG_7252_A	TOG_7252_A	O. glaberrima	*O. glaberrima*	NA	AR_glab	O. glaberrima
TOG_7400	TOG_7400	O. glaberrima	*O. glaberrima*	NA	AR_glab	O. glaberrima
TOG_7442_B	TOG_7442_B	O. glaberrima	*O. glaberrima*	NA	AR_glab	O. glaberrima
TOG_8347	TOG_8347	O. glaberrima	*O. glaberrima*	NA	AR_glab	O. glaberrima

Table S4. Accessions used for checking *RAE3* deletion corresponding with awn phenotype.

number in Fig. S11c	Barcode	accession_name	species	ID#	awn phenotype
1	RLT12537	O.barthii	*O.barthii*	IRGC104140	+
2	RLT12538	O.barthii	*O.barthii*	IRGC104983	+
3	RLT12539	WAB 010850	*O.barthii*	IRGC86524	+
4	RLT12540	W1468	*O.barthii*	IRGC101196	+
5	RLT12541	O.barthii	*O.barthii*	IRGC106291	+
6	RLT12542	O.barthii	*O.barthii*	IRGC104119	+
7	RLT12543	O.barthii	*O.barthii*	IRGC100933	+
8	RLT12544	O.barthii	*O.barthii*	IRGC100941	+
9	RLT12545	ZAKI BIAM-YANDE(WILD)1	*O.glaberrima*	TOG6193	-
10	RLT12546	YANDEV(1)	*O.glaberrima*	TOG5949	-
11	RLT12547	TOG5286	*O.glaberrima*	TOG5286	-
12	RLT12548	YAR KARENGESHE	*O.glaberrima*	TOG5440	-
13	RLT12549	SHENDAM (WEEDY)1	*O.glaberrima*	TOG5984	-
14	RLT12550	SHAWHON (2)	*O.glaberrima*	TOG5747	-
15	RLT12551	YAR BUTUKA	*O.glaberrima*	TOG5467	-
16	RLT12552	NEW AYOMA LOCAL (2)	*O.glaberrima*	TOG7402	-

Table S5. Primers used in this study.

Purpose	Primer Name	Primer Sequence	Description	Amplicon size (bp)
Positional cloning of *RAE3*	NK337/338_F	AAGACTTTCTTTTGCCGGAC	Physical location: chr6: 26425076	119
	NK337/338_R	TGGGCTTTGATGAATGAAGT	Physical location: chr6: 26425195	
	NK339/340_F	TAGGTAGGAGTAGGCCGGAT	Physical location: chr6: 27612992	124
	NK339/340_R	GCATGCACATATGTCACTGTGTAA	Physical location: chr6: 27613116	
	NK341/342_F	CGATCTCCTTTGCATCTTTC	Physical location: chr6: 28330534	118
	NK341/342_R	GGTGGTTAGCACCTTTGTGT	Physical location: chr6: 28330652	
	NK345/346_F	CTCATGCACAAATCATTCCA	Physical location: chr6: 28644007	112
	NK345/346_R	AGCCTGTGCACAAGTTCAG	Physical location: chr6: 28644119	
	NK369/370_F	GTTTCCAGATGCTGTCCATT	Physical location: chr6: 28804596	289
	NK369/370_R	GCACTTACCAGGTTCCATTG	Physical location: chr6: 28804885	
	NK347/348_F	CTCCTCTGATCACCTCGCT	Physical location: chr6: 28941431	130
	NK347/348_R	GGAGAGGAGCAGCTTCTTG	Physical location: chr6: 28941561	
	KG29051_F	CGTGTGTTTAGTGTGAAATTTTTCTAT	Physical location: chr6: 29050445	127
	KG29051_R	AGCCGCACAGAAAAGTGATT	Physical location: chr6: 29050561	
	KG29069_F1	TGCCATCCAATTATCAAACAA	Physical location: chr6: 29069041	60
	KG29069_R1	TGTTTATACAAGAAAAAATAAGGTTGG	Physical location: chr6: 29069107	
	KG29074_F	CATTGCACTAGCTTTAGACCTTGTTT	Physical location: chr6: 29074227	120
	KG29074_R	CCAAATTCTTATCTACTCGCTACTCA	Physical location: chr6: 29074299	
	KG29075_F	GATCGTCTAAAACTTACTCGTTACTG	Physical location: chr6: 29074907	62
	KG29075_R	TGAGATTACCAAGATCCTGTCC	Physical location: chr6: 29074965	
	KG29143_F2	ATTCCCTTTGTGCGTGTGTT	Physical location: chr6: 29143441	54
	KG29143_R2	TGGTTGAGCCAAAAGATGAA	Physical location: chr6: 29143494	
	KG29180_F	GCAGTGACGCAAGAAAGG	Physical location: chr6: 29175610	55
	KG29180_R	TCTCTTTTCCTACCGCCTTG	Physical location: chr6: 29175664	
	NK357/358_F	ACTCGTCGTCCACCTCAC	Physical location: chr6: 29231067	319
	NK357/358_R	TGGTTGGGACGCTAGTTATC	Physical location: chr6: 29231386	
	6KG29384_F	GTTTTGCTCAGGCAAATGAT	Physical location: chr6: 29384098	163
	6KG29384_R	GCACCCAAGATTTTATTGGA	Physical location: chr6: 29384261	
	RM20699_f	CCCGAGCCAGACAACATTCC	Physical location: chr6: 30327249	137
	RM20699_R	GAGGTGTGAGGTGAGGAAGATGC	Physical location: chr6: 30327386	
	RM5463_F	AGAATACTTTTACCACCCTTG	Physical location: chr6: 30986006	137
	RM5463_R	GTTTAATTAATCCAGGTAGGTCCT	Physical location: chr6: 30986143	
Screening of BAC and subclones	Awn291_F	CGTGTGTTTAGTGTGAAATTTTTCTAT	screening for BAC including 92-kb candidate region	116
	Awn291-R	AGCCGCACAGAAAAGTGATT	screening for BAC including 92-kb candidate region	
	Awn292_F	TGCATATGGCCATCCAAATA	screening for BAC including 92-kb candidate region	114
	Awn292_R	TCCATACGATGTTTAGTATACCTCCA	screening for BAC including 92-kb candidate region	
	KM1	CCGTCACGCAGAAGCTCAAG	Subclone 2-04C	126
	KM2	CGGAGTCGATGGCATCAAGT	Subclone 2-04C	
	KG29069_F2	CAATTATCAAACAAACAGAGAAACA	Subclone 2-03H	60
	KG29069_R2	TTGTTTATACAAGAACAATAAGGTTGG	Subclone 2-03H	
	KM3	GATGGTTGTGGTTGCTGCCTTC	Subclone 2-08A	239
	KM4	GTGTGTGGTGTAACTGTGTCGGAG	Subclone 2-08A	
	KG29143_F2	ATTCCCTTTGTGCGTGTGTT	Subclone 2-08G	53
	KG29143_R2	TGGTTGAGCCAAAAGATGAA	Subclone 2-08G	
	KM5	GGAACTAAACTCGGCCCAAG	Subclone 2-11F, 2-09G	120
	KM6	GAGTAGCACTAGCAGGACTTACTAGC	Subclone 2-11F, 2-09G	
Making constructs	KM49	cgggatccATGGAGGAAGATTCTGGTAG	cloning of *Os06g695600*, BamHI (underlined)	
	KM50	cccaagcttTCATGTGCCCCCCTTGT	cloning of *Os06g695600*, HindIII (underlined)	
	KM51	cgggatccATGGCGACACGGCCGTC	cloning of *Os06g695700*, BamHI (underlined)	
	KM52	cccaagcttTCAGCTCGATTTTCCCG	cloning of *Os06g695700*, HindIII (underlined)	
	KM83	cgagatctATGCGTCGTCTGGGACT	cloning of *Os06g695800*, BglII (underlined)	
	KM84	ccgtgatcaTCAGCTGAGCTCGAGGA	cloning of *Os06g695800*, SpeI (underlined)	
	KM55	cgggatccATGGAGCCGTCGCGGC	cloning of *Os06g695900*, BamHI (underlined)	
	KM56	cccaagcttCTAGGTGCTAGGGCCGT	cloning of *Os06g695900*, HindIII (underlined)	
	RAE3_pBS cloning_F	acagtctagacccgggATGGAGCCGTCGCGGCGGC	making RAE3(C136S)/ΩpCAMBIA1380 by NEBuilder	
	RAE3_pBS cloning_F	GGTGGACTCCTCTTAACTAGGTGCTAGGGCCGTTA	making RAE3(C136S)/ΩpCAMBIA1380 by NEBuilder	
	KM161	TGCCGAAATcCCGCCATG	RAE3(C136S)_site directed mutagenesis by PCR, F	
	KM162	CATGGCGGgATTTCGGCA	RAE3(C136S)_site directed mutagenesis by PCR, R	
	OsRAE3_FLAG_F	caaaagctttATGGAGCCGTCGCGGCGGCT	Making RAE3(WT)/ACTINproFLAGpCAMBIA1380, HindIII and SpeI, F	
	OsRAE3_FLAG_R	cttactagtCTAGGTGCTAGGGCCGTTAAG	Making RAE3(WT)/ACTINproFLAGpCAMBIA1380, HindIII and SpeI, R	
	KM154	CTGCCGAAATcaCGCCATGGGTTCCACGTC	Making RAE3(C136S), F	
	KM155	ATTTCGGCAGCAGCCGGAGC	Making RAE3(C136S), R	
	KM152	gagtctagaATGGAGCCGTCGCGGC	Os06g695900_CDS_F+Xba1	
	KM153	ctgggatccCTAGGTGCTAGGGCCGT	Os06g695900_CDS_R+BamH1	
	KM163	cgggatccatgTACGCGAAGCACTGCAAGC	OsRAE3ΔN_F+ATG+BamH1	
	KM164	catgaattcCTAGGTGCTAGGGCCGT	OsRAE3ΔN_R+EcoR1	
RAE3 expression analysis & cell localization	KM152	gagtctagaATGGAGCCGTCGCGGC	cloning of *Os06g695900* into pA7 (35S::MCS::GFPvector), XbaI (underlined)	
	KM153	ctgggatccGGTGCTAGGGCCGTTAAG	cloning of *Os06g695900* into pA7 (35S::MCS::GFPvector), BamHI (underlined)	
	UBQ5 RT-f	AAACCCTAACGGGGAAGACCATAA	Pf, qRT-PCR of ubiquitin (*Os01g0328400*) as internal control	
	UBQ5 RT-r	CCACAGTAATGGCGATCAAAATGA	Pr, qRT-PCR of ubiquitin (Os01g0328400) as internal control	
	KM145	CGAAGCAGCGGTGGAGCGA	Pf, qRT-PCR of RAE3	
	KM146	CTGCCGTTGCCGGAGTGGAC	Pr, qRT-PCR of RAE3	
	KBU63	caccATGGAGCCGTCGCGGC	Pf, cloning of Os/OgRAE3	
	KB112	GGTGCTAGGGCCGTTAAGGGC	Pr, OsRAE3 without stop	
	KBU59	CTGCCGAAATcCCGCCATGGGTTCCA	Pf, C136S_snp_circulated PCR (WT to C136S)	
	KBU60	CCCATGGCGGgATTTCGGCAGCAGCC	Pr, C136S_snp_circulated PCR (WT to C136S)	
AID experiment in yeast	KM115	CTactagtatgTACCCATACGATGTTCCTGACTATG	SpeI+atg_3×HA_AtIAA17_F, AtIAA17 cloning	
	KM129	CCCaagcttTCAAGCTCTGCTCTTGCACTTC	3×HA_AtIAA17+HindIII_R, AtIAA17 cloning	
	KM117	CTactagtTACGCGAAGCACTGCAAGC	SpeI+OsRAE3(ΔNΔC)_F	
	KM130	CGggatccGTCCTCGGGGTCGACGC	OsRAE3(ΔNΔC)+BamHI_R	
	KM119	CTggatccGAGAGGCTGAGCCGCCG	BamHI+OsTIR1(Δfbox)_F	
	KM137	CTgaattcCTATAGGATTTTAACAAAATTTGG	OsTIR1(Δfbox)_R+EcoR1	
	KM127	CTATGCACCCACACTTGATG	Sequence of OsTIR1Δ from forward	
	KM128	CAAGCCTTTGTCCGAGATAC	Sequence of OsTIR1Δ from reverse	
	KM134	GCATTAGAAAGCTCATCGTAGC	Sequence of 3HA_AtIAA17 from reverse	
	KBU73	CAACTCCAAGCTGGCCGCTCTAGAATGGACTACAA	Pf, around FLAG, for sequence	
	KBU69	ccgcgtcgaccccgaggacATGAGTAAAGGAGAAGAAC	Pf, cloning of GFP with BamHI within pOK833, NEBuilder	
	KBU70	cgcggcggctcagcctctcTAGTTCTACCATGCCATG	Pr, cloning of GFP with BamHI within pOK833, NEBuilder	
	KBU71	ttttgttaaaatcctatcgATGAGTAAAGGAGAAGAAC	Pf, cloning of GFP with EcoRI within pOK833, NEBuilder	
	KBU72	tatcgataagcttgatatcgTTATAGTTCTACCATGCCATG	Pr, cloning of GFP with EcoRI within pOK833, NEBuilder	
	KM135	CACAGACGTGGATGAAGTATC	Sequence of ScDma1ΔRING from reverse	
	KM136	GAAGATGTGTCGTTCCAGTG	Sequence of Ucc1ΔFbox from reverse	

*Physical localization is based on The Rice Annotation Project (https://rapdb.dna.affrc.go.jp/index.html)

Dataset S1 (separate file).

Haplotype information detected by the nucleotide diversity analysis.

SI References

1. R. A. Shim, E. R. Angeles, M. Ashikari, T. Takashi, Development and evaluation of *Oryza glaberrima* Steud. chromosome segment substitution lines (CSSLs) in the background of O. sativa L. cv. Koshihikari. *Breed. Sci.* **60**, 613–619 (2010).
2. H. Sakai, *et al.*, Rice Annotation Project Database (RAP-DB): An Integrative and Interactive Database for Rice Genomics. *Plant and Cell Physiology* **54**, e6–e6 (2013).
3. Y. Kawahara, *et al.*, Improvement of the *Oryza sativa* Nipponbare reference genome using next generation sequence and optical map data. *Rice* **6**, 4 (2013).
4. K. L. Howe, *et al.*, Ensembl Genomes 2020—enabling non-vertebrate genomic research. *Nucleic Acids Research* **48**, D689–D695 (2020).
5. H. Li, Aligning sequence reads, clone sequences and assembly contigs with BWA-MEM (figshare, 2014).
6. H. Li, *et al.*, The Sequence Alignment/Map format and SAMtools. *Bioinformatics* **25**, 2078–2079 (2009).
7. J. T. Simpson, *et al.*, ABySS: A parallel assembler for short read sequence data. *Genome Res.* **19**, 1117–1123 (2009).
8. J. G. Ruby, P. Bellare, J. L. DeRisi, PRICE: Software for the Targeted Assembly of Components of (Meta) Genomic Sequence Data. *G3 Genes|Genomes|Genetics* **3**, 865–880 (2013).
9. M. Boetzer, C. V. Henkel, H. J. Jansen, D. Butler, W. Pirovano, Scaffolding pre-assembled contigs using SSPACE. *Bioinformatics* **27**, 578–579 (2011).
10. J. C. Abbott, D. M. Aanensen, K. Rutherford, S. Butcher, B. G. Spratt, WebACT--an online companion for the Artemis Comparison Tool. *Bioinformatics* **21**, 3665–3666 (2005).
11. T. J. Carver, *et al.*, ACT: the Artemis comparison tool. *Bioinformatics* **21**, 3422–3423 (2005).
12. L. Xia, *et al.*, Rice Expression Database (RED): An integrated RNA-Seq-derived gene expression database for rice. *Journal of Genetics and Genomics* **44**, 235–241 (2017).
13. Y. Zhang, *et al.*, A highly efficient rice green tissue protoplast system for transient gene expression and studying light/chloroplast-related processes. *Plant Methods* **7**, 30 (2011).
14. D. Mumberg, R. Müller, M. Funk, Yeast vectors for the controlled expression of heterologous proteins in different genetic backgrounds. *Gene* **156**, 119–122 (1995).
15. K. Nishimura, T. Fukagawa, H. Takisawa, T. Kakimoto, M. Kanemaki, An auxin-based degron system for the rapid depletion of proteins in nonplant cells. *Nat Methods* **6**, 917–922 (2009).
16. K. Obara, *et al.*, Proteolysis of adaptor protein Mmr1 during budding is necessary for mitochondrial homeostasis in Saccharomyces cerevisiae. *Nat Commun* **13**, 2005 (2022).
17. M. Wang, *et al.*, The genome sequence of African rice (Oryza glaberrima) and evidence for independent domestication. *Nat Genet* **46**, 982–988 (2014).
18. The 3,000 rice genomes project, The 3,000 rice genomes project. *GigaSci* **3**, 7 (2014).

19. G. Melandri, *et al.*, Multiple Small-Effect Alleles of Indica Origin Enhance High Iron-Associated Stress Tolerance in Rice Under Field Conditions in West Africa. *Front. Plant Sci.* **11**, 604938 (2021).
20. J. Duitama, *et al.*, Whole Genome Sequencing of Elite Rice Cultivars as a Comprehensive Information Resource for Marker Assisted Selection. *PLoS ONE* **10**, e0124617 (2015).
21. X. Xu, *et al.*, Resequencing 50 accessions of cultivated and wild rice yields markers for identifying agronomically important genes. *Nat Biotechnol* **30**, 105–111 (2012).
22. R. S. Meyer, *et al.*, Domestication history and geographical adaptation inferred from a SNP map of African rice. *Nat Genet* **48**, 1083–1088 (2016).
23. H. Li, R. Durbin, Fast and accurate short read alignment with Burrows-Wheeler transform. *Bioinformatics* **25**, 1754–1760 (2009).
24. H. Li, R. Durbin, Fast and accurate long-read alignment with Burrows–Wheeler transform. *Bioinformatics* **26**, 589–595 (2010).
25. A. McKenna, *et al.*, The Genome Analysis Toolkit: A MapReduce framework for analyzing next-generation DNA sequencing data. *Genome Res.* **20**, 1297–1303 (2010).
26. M. A. DePristo, *et al.*, A framework for variation discovery and genotyping using next-generation DNA sequencing data. *Nat Genet* **43**, 491–498 (2011).
27. G. A. Auwera, *et al.*, From FastQ Data to High-Confidence Variant Calls: The Genome Analysis Toolkit Best Practices Pipeline. *Current Protocols in Bioinformatics* **43** (2013).
28. R. Poplin, *et al.*, Scaling accurate genetic variant discovery to tens of thousands of samples. *bioRxiv* (2017) https:/doi.org/10.1101/201178 (February 25, 2022).
